# Impaired cortical development and translational control in a missense mouse model of *DDX3X* syndrome

**DOI:** 10.1242/dmm.052498

**Published:** 2025-11-28

**Authors:** Abigail J. Poff, Nicole D. Moss, Debra L. Silver

**Affiliations:** ^1^ Department of Molecular Genetics and Microbiology, Duke University Medical Center, Durham, NC 27710, USA; ^2^ Department of Neurobiology, Duke University Medical Center, Durham, NC 27710, USA; ^3^ Department of Cell Biology, Duke University Medical Center, Durham, NC 27710, USA; ^4^ Duke Institute for Brain Sciences and Duke Regeneration Center, Duke University Medical Center, Durham, NC 27710, USA

**Keywords:** *DDX3X*, Corticogenesis, Radial glia, Translation

## Abstract

Heterozygous mutations in the X-linked RNA helicase *DDX3X* cause *DDX3X* syndrome, a rare neurodevelopmental disorder associated with cortical malformations and autism spectrum disorder. Among ∼200 known *DDX3X* variants, half are missense, while the remainder are predicted loss-of-function (LoF) variants. LoF mouse models reveal that *Ddx3x* controls progenitors' ability to generate excitatory neurons. Yet, how missense mutations impact corticogenesis *in vivo* is unknown. Here, we generated a conditional mouse model of *DDX3X^T532M^*, a clinically severe and recurrent *DDX3X* syndrome variant found in affected females. Using *Emx1*-Cre-mediated expression of *Ddx3x^T532M^* in cortical progenitors, we showed that *Ddx3*x*^T532M^* alters corticogenesis. *Ddx3x^T532M^* conditional hemizygous males have severe microcephaly and apoptosis. In contrast, *Ddx3x^T532M^* conditional heterozygous (cHet) females exhibit mild reductions in cortical size and neurogenesis. Using polysome fractionation of *Ddx3x^T532M^* and *Ddx3x^LoF^* cHet female cortices, we discovered that *Ddx3x^T532M^* affects translation, with *Ddx3x^T532M^* cHet females showing qualitative differences from *Ddx3x^LoF^* cHet females. Collectively, these findings suggest that although *Ddx3x^T532M^* and *Ddx3x^LoF^* have similar impacts on corticogenesis in cHet females, they have distinct molecular targets. Our study establishes a new *in vivo* model for understanding the etiology of *DDX3X* syndrome.

## Teaser

**Summary:** We generated and characterized a new mouse model for *DDX3X* syndrome. We show that the missense variant *Ddx3x^T532M^* impacts cortical development and provide new insights into DDX3X molecular targets.

## INTRODUCTION

Heterozygous *de novo* mutations in the X-linked RNA helicase *DDX3X* cause the neurodevelopmental disorder *DDX3X* syndrome. Individuals with *DDX3X* syndrome present with varying degrees of intellectual disability, developmental delay, autism spectrum disorder and structural brain malformations (Lennox et al., 2020; Snijders Blok et al., 2015; Dai et al., 2022; Wang et al., 2018; Parra et al., 2024; Levy et al., 2023; Beal et al., 2019; Tang et al., 2021). Clinical, imaging and genetic data from 107 individuals with *DDX3X* syndrome indicate that clinical severity is associated with mutation type (Lennox et al., 2020). For example, individuals with specific missense variants have clinically severe presentations, whereas those with other missense variants are associated with mild outcomes resembling loss of function (LoF). Clinically severe presentations include polymicrogyria (PMG), increased prevalence and severity of corpus callosum malformations, epilepsy and cardiac malformations (Lennox et al., 2020). In contrast, patients with clinically mild presentations show fewer corpus callosum alterations and no PMG. This genotype–phenotype association suggests that distinct pathogenic mechanisms are associated with distinct *DDX3X* missense variants.

**Fig. 1.**
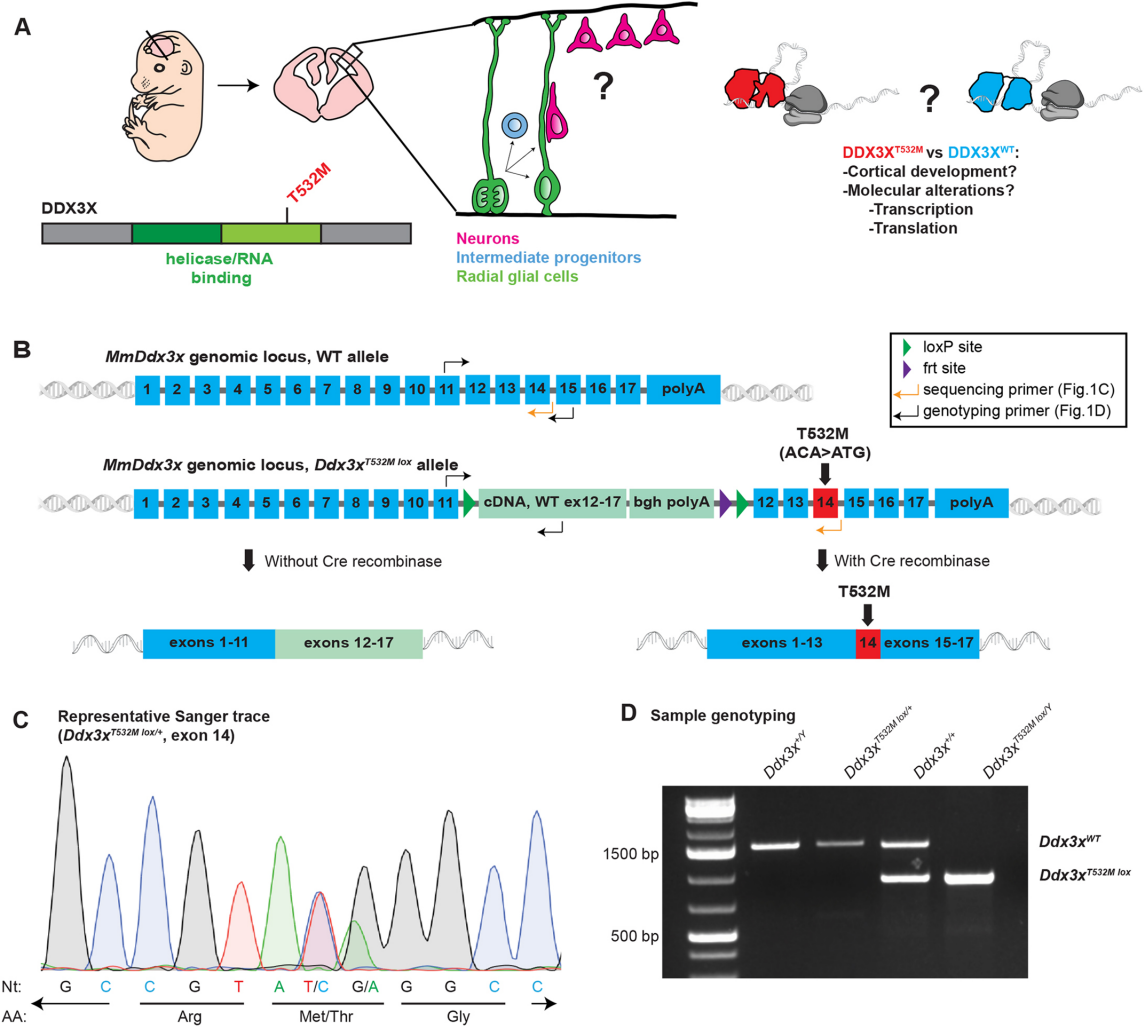
**Generation of conditional knock-in model for *Ddx3x^T532M^*.** (A) Overview of project aims and cortical development. Ribosome large subunit, small subunit and ssRNA brush illustrations from NIH BioArt Source (see Materials and Methods). Not to scale. (B) Schematic of *Ddx3x^T532M lox^* conditional allele design. Minigene consists of green-shaded boxes; boxes shaded blue are within endogenous intron/exon context. Red exon 14 indicates T532M mutation. Dark-green triangles represent loxP sites; purple triangle represents remaining *frt* site from excision of *frt*-flanked neomycin selection cassette used for initial recombinant selection. Sequencing primer is shown as an orange arrow; genotyping primers are denoted by black arrows. DNA and RNA illustrations from NIH BioArt Source (see Materials and Methods). Not to scale. (C) Representative trace of sequencing across exon 14 in genomic DNA from a *Ddx3x^T532M lox/+^* female mouse, showing overlapping traces of nucleotides (nt) of the two alleles (ACA/ATG), and their resulting amino acids (AA). This assay used intronic primers to exclude amplification of the exon 14 contained within the minigene construct. (D) Representative agarose gel image results of DNA genotyping of denoted genotypes using primers from Fig. 1B.

*DDX3X* encodes an RNA-binding protein of the DEAD-box helicase family, consisting of two helicase/RNA binding domains flanked by low-complexity N-terminal and C-terminal domains (Fig. 1A) (Sharma and Jankowsky, 2014). Although broadly implicated in mRNA metabolism, DDX3X is best characterized as a translational regulator (Lai et al., 2008; Shih et al., 2008). It associates with transcript 5′ untranslated regions (UTRs) to resolve secondary structures and promote translation initiation (Calviello et al., 2021; Chen et al., 2018; Phung et al., 2019). DDX3X is also a component of RNA–protein granules (Hondele et al., 2019; Elvira et al., 2006; Kanai et al., 2004; Lennox et al., 2020; Markmiller et al., 2018; Mosti et al., 2025).

Both clinical and murine model data indicate that development of the cerebral cortex is impacted by *DDX3X* mutation (Hoye et al., 2022; Lennox et al., 2020; Boitnott et al., 2021). Corticogenesis initiates when neuroepithelial cells produce key neurogenic and gliogenic cortical progenitors, termed radial glial cells (RGCs), at the ventricular zone (Gotz and Huttner, 2005). During neurogenesis, RGCs undergo both symmetric and asymmetric divisions to produce RGCs, excitatory neurons and neurogenic intermediate progenitors (IPs) (Fig. 1A) (Taverna et al., 2014; Malatesta et al., 2000; Noctor et al., 2001). Newborn neurons then migrate into the cortical plate in an inside-out laminar pattern, with deep-layer neurons born earlier and superficial neurons born later (Polleux et al., 1997; McConnell and Kaznowski, 1991). Cortex-specific *Ddx3x* LoF in female mice causes extensive apoptosis and microcephaly (Hoye et al., 2022). In contrast, cortex-specific *Ddx3x* haploinsufficiency in female mice and LoF in male mice results in milder phenotypes, with relatively normal brain size but imbalanced cell fate decisions toward progenitor self-renewal over neuronal production. In post-mitotic neurons, reduction of *Ddx3x* levels alters neurite outgrowth (Chen et al., 2016b), dendritogenesis and dendritic spine development (Mossa et al., 2025). Further, germline *Ddx3x* haploinsufficiency causes developmental delays, as well as behavioral changes in adulthood, including hyperactivity and anxiety-like phenotypes (Boitnott et al., 2021), some of which are recapitulated in a cortex-specific haploinsufficiency model (Mossa et al., 2025). These studies highlight essential requirements for *Ddx3x* in cortical development and function.

Recent *in vitro* studies have begun to elucidate cellular and molecular mechanisms of *DDX3X* missense variants. Expression of clinically severe and mild missense *DDX3X* mutations in murine primary cortical progenitors differentially impairs neurogenesis and neuronal survival (Mosti et al., 2025). These same mutations alter DDX3X protein binding partners, subcellular localization and transcriptomic state of cortical cells. Additionally, severe *DDX3X* missense mutants lack RNA helicase function, associated with reduced translation of target mRNAs (Calviello et al., 2021; Lennox et al., 2020; Owens et al., 2024). However, the molecular and cellular impact of *DDX3X* missense variants *in vivo* remains unknown.

*DDX3X^T532M^* is a recurrent mutation found in six female patients with *DDX3X* syndrome to date (Lennox et al., 2020; Parra et al., 2024; Dai et al., 2022). Four of these individuals presented with concordant severe neuroanatomical findings, including PMG, thin corpus callosum and microcephaly (Lennox et al., 2020); one individual presented with mild neuroanatomical defects, notably lacking PMG, while the remaining individual's clinical report did not include neuroimaging to assess brain architecture (Dai et al., 2022; Parra et al., 2024). Like most missense variants, *DDX3X^T532M^* is located within one of the protein's two helicase/RNA-binding domains. Additionally, biochemical analyses demonstrate that DDX3X^T532M^ is unable to resolve dsRNA duplexes, and *in vitro* studies show that DDX3X^T532M^ exhibits altered subcellular localization and impaired neurogenesis (Mosti et al., 2025; Lennox et al., 2020). Thus, *Ddx3x^T532M^* is an ideal candidate to understand pathogenic mechanisms of clinically severe missense mutations.

In this study, we generated a Cre inducible conditional mouse model for *Ddx3x^T532M^*, which we used to assess cortical development (Fig. 1A). We demonstrated that *Ddx3x^T532M^* mRNA and protein are expressed in the cortex following Cre induction, establishing the validity of this disease model. *Emx1*-Cre;*Ddx3x^T532M lox/+^* conditional heterozygous (cHet) females largely phenocopy *Ddx3x* haploinsufficiency, with slightly reduced cortical size and mild neurogenesis phenotypes. In contrast, *Emx1*-Cre;*Ddx3x^T532M lox/Y^* conditional hemizygous (cHemi) males exhibit microcephaly and apoptosis. Finally, we employed polysome profiling to discover transcriptional and translational alterations resulting from *Ddx3x^T532M^* expression in the developing cortex. Our study expands our understanding of *DDX3X* biology and establishes a new missense mouse model to understand *DDX3X* syndrome.

## RESULTS

### *Ddx3x^T532M lox^* conditional model generation and validation

To understand how the clinically severe missense variant *Ddx3x^T532M^* affects cortical development *in vivo*, we generated a conditional knock-in mouse model (Fig. 1B). First, we inserted a wild-type minigene for *Ddx3x* exons 12-17, flanked by loxP sites, between the endogenous exons 11 and 12 of *Ddx3x* in mouse embryonic stem (ES) cells. We also altered the endogenous exon 14 to encode T532M by mutating two base pairs within an ACA codon for threonine to ATG for methionine (Fig. 1B). Of note, humans use an alternate ACG codon for threonine, and thus a single nucleotide (C>G) mutation causes the *DDX3X^T532M^* variant in patients with *DDX3X* syndrome. Once validated, these targeted ES cells were microinjected into embryos at the blastocyst stage to create chimeras. Successful knock-in of these sequences was confirmed by Sanger sequencing across the modified *Ddx3x* locus (Fig. 1C). Chimeric mice with germline transmission of the modified allele, which is hereafter referred to as *Ddx3x^T532M lox^*, were used as founders and bred on a C57BL/6J background.

**Fig. 2.**
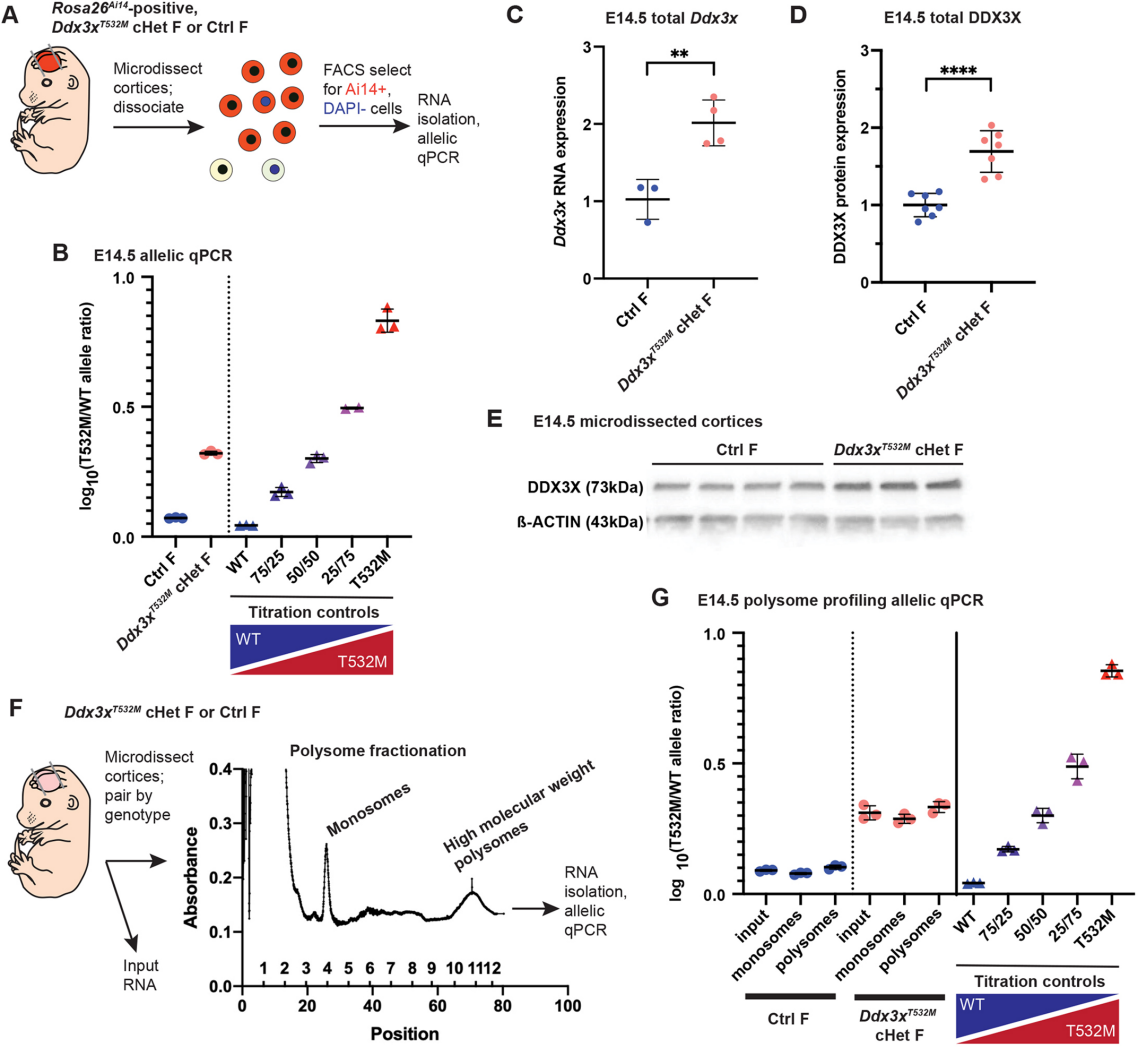
**Validation of conditional knock-in model for *Ddx3x^T532M^*.** (A) Schematic of fluorescence-activated cell sorting (FACS) isolation of Cre^+^ cells. (B) Allelic quantitative reverse transcription PCR (qRT-PCR) analysis at E14.5 plotted as the ratio of the two alleles and normalized to 50/50 T532M/wild-type (WT) titration controls; *n*=3 embryos/genotype from three litters. Each point represents one embryo, assayed in triplicate and averaged. Results are representative of two quantitative PCR (qPCR) experiments performed. (C) Total *Ddx3x* qPCR of FACS-isolated Cre^+^ cells at embryonic day (E)14.5. Each point represents an embryo, assayed in triplicate and averaged. *n*=3 *Emx1-*Cre;*Rosa26^Ai14^*;*Ddx3x^+/+^* (Ctrl, control) female embryos; *n*=4 *Emx1-*Cre;*Rosa26^Ai14^*;*Ddx3x^T532M lox/+^* conditional heterozygous (cHet) female embryos, from three litters. RNA levels were normalized to β-actin. Results are representative of two qPCR experiments performed. (D) Quantification of E14.5 DDX3X protein expression normalized to ACTB. Each point represents an embryo. *n*=7 embryos/genotype from five litters. Results are representative of two western blots performed. (E) Representative western blot for total DDX3X, with ACTB as a loading control, in E14.5 microdissected cortices of denoted genotypes. Results are representative of two western blots performed. (F) Schematic of polysome profiling approach used to assess translation of *Ddx3x* alleles. (G) Allelic qRT-PCR analysis at E14.5 from polysome fractionation samples, plotted as the ratio of the two alleles and normalized to 50/50 T532M/WT titration controls. Each point represents a single sample (composed of cortices from a pair of like-genotype embryos), assayed in triplicate and averaged. qPCR was performed once. *n*=3 samples/genotype from five litters. **P<0.005; ****P<0.0001. Statistics are Student's unpaired, two-tailed *t*-test (C,D). Error bars, mean±s.d.

Having generated and sequence confirmed this model, we next induced expression of *Ddx3x^T532M^* specifically in the developing cerebral cortex. We used the *Emx1*-Cre driver to induce recombination at embryonic day (E)9.5 in RGCs and their progeny (Gorski et al., 2002) (Fig. 1A,B). For this and subsequent studies, we evaluated *Emx1*-Cre;*Ddx3x^T532M lox/+^* female mice (referred to as *Ddx3x^T532M^* cHet females) to model *de novo* mutations in *DDX3X* syndrome, as this clinical variant has only been reported in affected females*.* To validate and quantify *Ddx3x^T532M^* expression *in vivo*, we used the fluorescent Cre reporter line, *Rosa26^Ai14^*, which expresses tdTomato in Cre-positive cells (Madisen et al., 2010) (Fig. 2A). Ai14-positive *Ddx3x^T532M^* cHet and littermate control female embryonic cortices were microdissected and dissociated into single-cell suspensions. Cre-positive live (tdTomato^+^DAPI^−^) cells were then isolated with fluorescence-activated cell sorting (FACS). To measure the ratio of *Ddx3x^WT^*:*Ddx3x^T532M^* expression in the cortex, we performed quantitative reverse transcription PCR (qRT-PCR) on cDNA from sorted cells with an allelic TaqMan assay capable of discriminating between the two mRNA alleles. To monitor allelic ratios, we generated titration controls using HEK-293T cells transfected with known ratios of murine *Ddx3x^WT^* and *Ddx3x^T532M^* (Fig. S1A,B). Importantly, at both E11.5 and E14.5, *Ddx3x^T532M^* cHet females expressed *Ddx3x^WT^* and *Ddx3x^T532M^* RNA in a 1:1 ratio (Fig. 2B; Fig. S1A,B). As expected, control females only expressed *Ddx3x^WT^*. This demonstrates that the *Ddx3x^T532M^* cHet female model performs as expected, with both *Ddx3x^WT^* and *Ddx3x^T532M^* alleles equivalently expressed in bulk cortical cells at E11.5 and E14.5.

We next measured total *Ddx3x* RNA and protein levels in the *Ddx3x^T532M^* cHet model. Compared to littermate female control cortices, *Ddx3x^T532M^* cHet female cortices showed a 2-fold increase in total *Ddx3x* RNA levels at E14.5 (Fig. 2C) and slightly increased (∼1.6-fold) protein levels as measured by western blotting (Fig. 2D,E). Notably, protein levels were unchanged at E12.5 (Fig. S1D,E). Given this observation, we assessed whether DDX3X^T532M^ and DDX3X^WT^ protein are translated equivalently at E14.5. For this, we used polysome profiling combined with allelic TaqMan qRT-PCR (Fig. 2F). Polysome profiling uses a sucrose gradient to separate mRNAs bound by several ribosomes (termed polysomes) or by only one ribosome (termed monosomes) (Arava et al., 2003). Thus, polysome profiling enabled us to assess translation of *Ddx3x* alleles based on their relative expression in various ribosome-associated fractions. To perform polysome profiling, we microdissected E14.5 *Ddx3x^T532M^* cHet and control female cortices, followed by lysate preparation, ultracentrifugation through a sucrose gradient and polysome fractionation. RNA extraction and subsequent allelic TaqMan quantitative PCR (qPCR) indicated that, in *Ddx3x^T532M^* cHet cortices, both *Ddx3x^WT^* and *Ddx3x^T532M^* RNAs are equally present in both monosomes and high-molecular-weight polysome fractions, interpreted relative to titration controls (Fig. 2G; Fig. S1F). Altogether, these data indicate that although there is a slight increase in overall levels of DDX3X protein in the cortices of *Ddx3x^T532M^* cHet female mice compared to those in the cortices of control female mice, both *Ddx3x^WT^* and *Ddx3x^T532M^* alleles are equivalently expressed at the RNA and protein level.

### Cortical development defects in *Emx1*-Cre;*Ddx3x^T532M^* mice

**Fig. 3.**
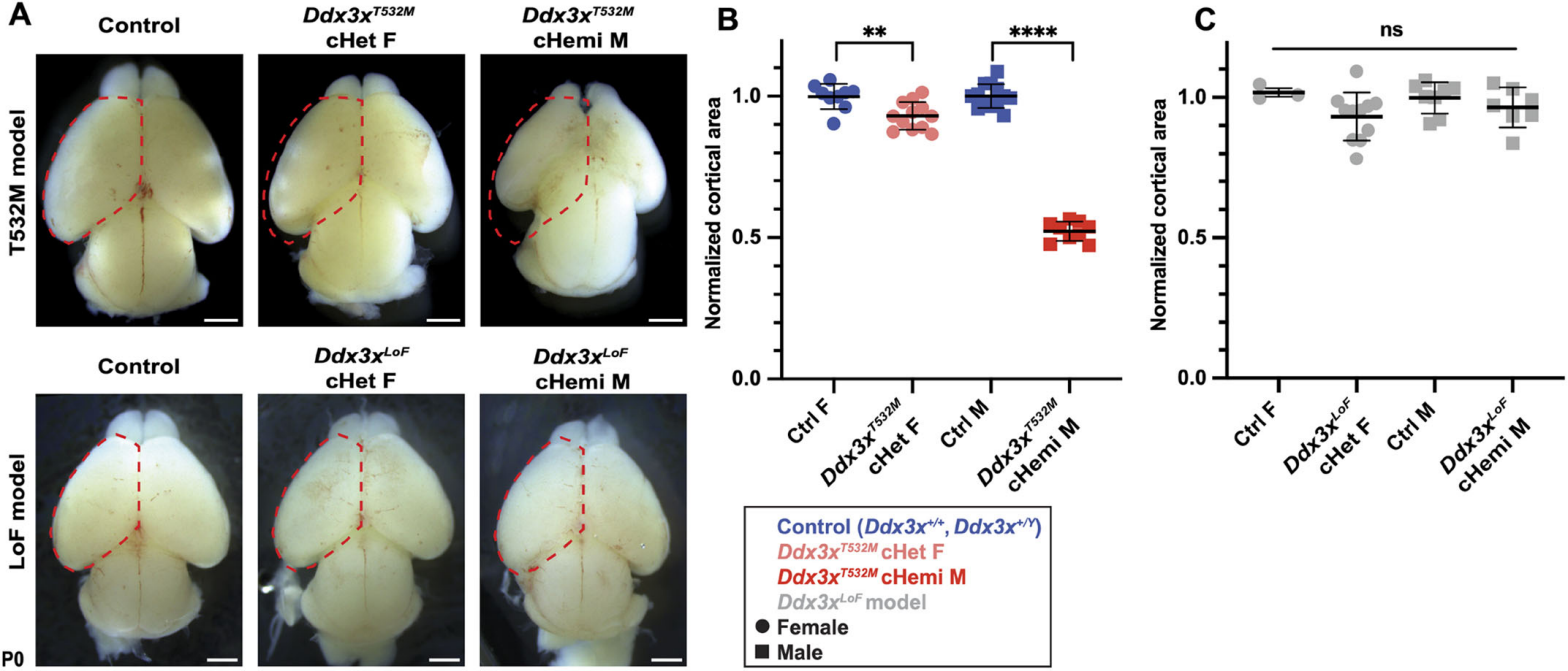
***Ddx3x^T532M^* conditional knock-in mice show sex differences in brain size.** (A) Representative images of whole brains of denoted genotypes at postnatal day (P)0. Red dashed lines outline cortex within each control, superimposed onto subsequent *Ddx3x* mutant brains. (B,C) Quantifications of total cortical area of denoted genotypes, with each litter normalized to the average cortical area of control littermates. Squares represent males, and circles represent females. Each point represents an individual embryo. For T532M, *n*=13 control males, including *Ddx3x^+/Y^* and *Emx1*-Cre;*Ddx3x^+/Y^*; *n*=9 control females, including *Ddx3x^+/+^* and *Emx1*-Cre;*Ddx3x^+/+^*; *n*=12 *Emx1*-Cre;*Ddx3x^T532M lox/+^* cHet females; *n*=9 *Emx1*-Cre;*Ddx3x^T532M lox/Y^* conditional hemizygous (cHemi) males from nine litters. For loss-of-function (LoF), *n*=3 *Ddx3x^lox/+^* control females, *n*=11 *Emx1-Cre;Ddx3x^lox/+^* cHet females, *n*=8 *Ddx3x^lox/Y^* control males, *n*=7 *Emx1-Cre;Ddx3x^lox/Y^* cHemi males, from four litters; although these shown were independently collected, cortical area from this model has also been previously published (Hoye et al., 2022). F, female; M, male. Statistics are one-way ANOVA with multiple comparisons. For LoF model, no pairwise comparison was statistically significant. For T532M, all pairwise comparisons with *Ddx3x^T532M^* cHemi males were significant (****P<0.0001), and the comparison between control and *Ddx3x^T532M^* cHet females was also significant (**P<0.005); all other comparisons were not significant (ns). Error bars, mean±s.d. Scale bars: 0.1 cm (A).

We next characterized the impact of *Ddx3x^T532M^* expression on overall brain size. We first assessed gross cortical morphology of *Emx1*-Cre;*Ddx3x^T532M lox/Y^* (*Ddx3x^T532M^* cHemi) male embryos, *Ddx3x^T532M^* cHet female embryos and control embryos of both sexes by whole-mount imaging of postnatal day (P)0 brains. *Ddx3x^T532M^* cHet females exhibited a mild 6% reduction in cortical area compared to control females (Fig. 3A,B). In contrast, *Ddx3x^T532M^* cHemi male mice expressing *Ddx3x^T532M^* from their sole X chromosome exhibited a 47% reduction in cortical area compared to that in male controls (Fig. 3A,B). A slight, but significant, 12% decrease in cortical area was also seen in Cre-negative *Ddx3x^T532M lox/Y^* males, with apparent reduced body size (Fig. S2A-C). Neither of these findings were evident in Cre-negative *Ddx3x^T532M lox/+^* females (Fig. S2A,B). We also observed reduced recovery of *Ddx3x^T532M lox/Y^* male mice, irrespective of Cre inheritance (Fig. S2D). This suggests that, in males, genomic modifications at the *Ddx3x^T532M^* locus induce a background phenotype reducing recovery of this genotype. Of note, this lethality is not due to microcephaly, as cortical loss across diverse *Emx1*-Cre models does not reduce viability (Lupan et al., 2023; Mao et al., 2015, 2016; Hoye et al., 2022; McMahon et al., 2014). Importantly, in subsequent experiments, we included Cre-negative controls to account for potential background effects of the modified allele.

We also compared the brain size of *Ddx3x^T532M^* cHet female and *Ddx3x^T532M^* cHemi male mice with the *Ddx3x* LoF model, including *Emx1*-Cre;*Ddx3x^lox/+^* (*Ddx3x^LoF^* cHet) females and *Emx1*-Cre;*Ddx3x^lox/Y^* (*Ddx3x^LoF^* cHemi) males. Consistent with published findings (Hoye et al., 2022), microcephaly was not observed in *Ddx3x^LoF^* cHet females and *Ddx3x^LoF^* cHemi males compared to control littermates (Fig. 3A-C). Thus, compared to *Ddx3x* haploinsufficiency, *Ddx3x^T532M^* cHet female mice have a very mild microcephaly, whereas *Ddx3x^T532M^* cHemi males have a profound microcephaly.

**Fig. 4.**
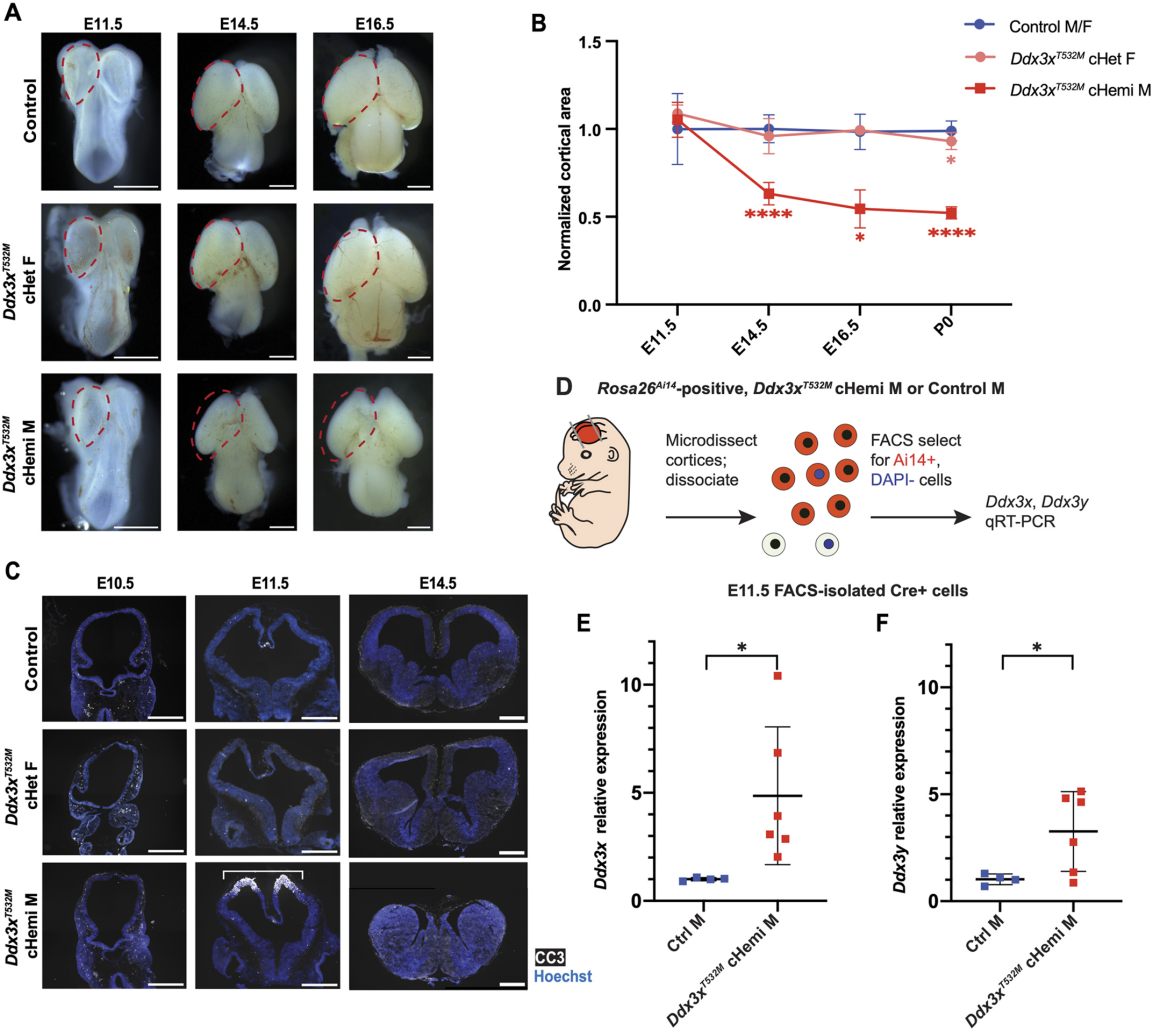
***Ddx3x^T532M^* cHemi males exhibit striking microcephaly, apoptosis and upregulation of both *Ddx3x* and *Ddx3y.*** (A) Representative whole-mount brain images of denoted genotypes and embryonic stages. Red dashed lines outline cortex in the control brain image for each stage, superimposed onto *Ddx3x^T532M^* cHet female and *Ddx3x^T532M^* cHemi male brains of the same stage. (B) Quantifications of total cortical area of denoted genotypes over embryonic time, with each litter normalized to the average cortical area of its control littermates. P0 quantifications re-plotted from Fig. 3. Traces are color coded by genotype (blue, control males/females; salmon, *Emx1*-Cre;*Ddx3x^T532M lox/+^* cHet females; red, *Emx1*-Cre;*Ddx3x^T532M lox/Y^* cHemi males). Any comparisons not shown are not significant. (C) Cleaved caspase 3 (CC3, white) and Hoechst (blue) immunofluorescence in coronal cryosections from embryonic brains of denoted stages and genotypes. Bracket in *Ddx3x^T532M^* cHemi M image at E11.5 outlines CC3^+^ cortex. Staining as shown is representative of two independent immunofluorescence experiments. The image for *Ddx3x^T532M^* cHemi M (E14.5) is also shown in Fig. S2. (D) Schematic of FACS approach to isolate Cre^+^ cells at E11.5. (E,F) qRT-PCR results for total *Ddx3x* expression (E) and total *Ddx3y* expression (F) in E11.5 FACS-isolated Cre^+^ samples from *Emx1*-Cre;*Rosa26^Ai14^*;*Ddx3x^+/Y^* control male and *Emx1*-Cre;*Rosa26^Ai14^*;*Ddx3x^T532M lox/Y^* cHemi male cortices. qRT-PCR results are first normalized to β-actin, then to control males. Each point is representative of a single embryo, assayed in triplicate. Points are color coded by genotype. *n*=4 *Emx1*-Cre;*Rosa26^Ai14^*;*Ddx3x^+/Y^* (control) males, *n*=6 *Emx1*-Cre;*Rosa26^Ai14^*;*Ddx3x^T532M lox/Y^* cHemi males from two litters. qPCR results representative of two qPCR experiments performed. Statistics assessed by one-way ANOVA with multiple comparisons, with only statistically significant comparisons shown (B), Student's unpaired, two-tailed *t*-test (E,F). *P<0.05; ****P<0.0001. Error bars, mean±s.d. Scale bars: 0.1 cm (A), 0.02 cm (C).

We next aimed to understand the developmental basis for the cortical size phenotypes of *Ddx3x^T532M^* cHet female and cHemi male mice. We determined the onset of the microcephaly phenotype by collecting and quantifying brains from both genotypes, as well as littermate controls, across embryonic timepoints spanning neurogenesis. At E11.5, E14.5 and E16.5, the embryonic cortical area of *Ddx3x^T532M^* cHet females was comparable to that of controls, consistent with their mild phenotype at P0 (Fig. 4A,B). In contrast, at E14.5, a significant 37% reduction in cortical area was apparent in *Ddx3x^T532M^* cHemi males compared to controls, ∼5 days after the onset of the cortex-specific Cre expression (Fig. 4A,B). As microcephaly is often associated with apoptotic cell death, we next assessed apoptosis in *Ddx3x^T532M^* cHemi males by immunostaining for cleaved caspase 3 (CC3). CC3 signal was not apparent in *Ddx3x^T532M^* cHemi male cortices at E10.5 but was obvious at E11.5 (Fig. 4C). In contrast, apoptosis was not apparent in *Ddx3x^T532M^* cHet females or littermate controls at any stage assessed (Fig. 4C), and was not present in *Ddx3x^T532M lox/Y^* male mice lacking a Cre driver (Fig. S2E). The lack of apoptosis in *Ddx3x^T532M^* cHet female mice is consistent with their normal brain size at these stages (Fig. 4A,B). Thus, we conclude that apoptotic cell death is associated with microcephaly specifically in *Ddx3x^T532M^* cHemi male mice.

We next aimed to understand whether altered expression of *Ddx3x* and its Y chromosome paralog, *Ddx3y*, could explain why *Ddx3x^T532M^* cHemi males have more severe phenotypes than those of *Ddx3x^T532M^* cHet females. Previous studies indicate that *Ddx3y* is transcriptionally upregulated in response to *Ddx3x* loss in mouse models and human cell culture, which may compensate for *Ddx3x* loss in control of brain size (Hoye et al., 2022; Rengarajan et al., 2025; Patmore et al., 2020; Szappanos et al., 2018). In addition, *Ddx3y* can rescue *Ddx3x* phenotypes in the hindbrain and in the hematopoietic system (Patmore et al., 2020; Szappanos et al., 2018). To test whether alterations in *Ddx3y* levels contribute to sexually dimorphic phenotypes in *Ddx3x^T532M^* mice, we measured *Ddx3y* levels in *Ddx3x^T532M^* cHemi males and littermate controls. We again used the *Rosa26^Ai14^* Cre reporter with FACS to isolate Cre-positive live cells from embryonic cortices at E11.5 (Fig. 4D). Compared to control cortices, *Ddx3x^T532M^* cHemi male cortices had higher *Ddx3x* expression (Fig. 4E). *Ddx3y* was also significantly increased in cortical cells from *Ddx3x^T532M^* cHemi males compared to those from controls (Fig. 4F). These data demonstrate that, as in *Ddx3x^LoF^* cHemi male cortices (Hoye et al., 2022), *Ddx3y* is transcriptionally upregulated in *Ddx3x^T532M^* cHemi male cortices. However, unlike *Ddx3x^LoF^* cHemi males, *Ddx3x^T532M^* cHemi males also upregulate *Ddx3x*. This dysregulation of both paralogs could contribute to the apoptosis and microcephaly phenotypes observed in cHemi male mice expressing *Ddx3x^T532M^*.

**Fig. 5.**
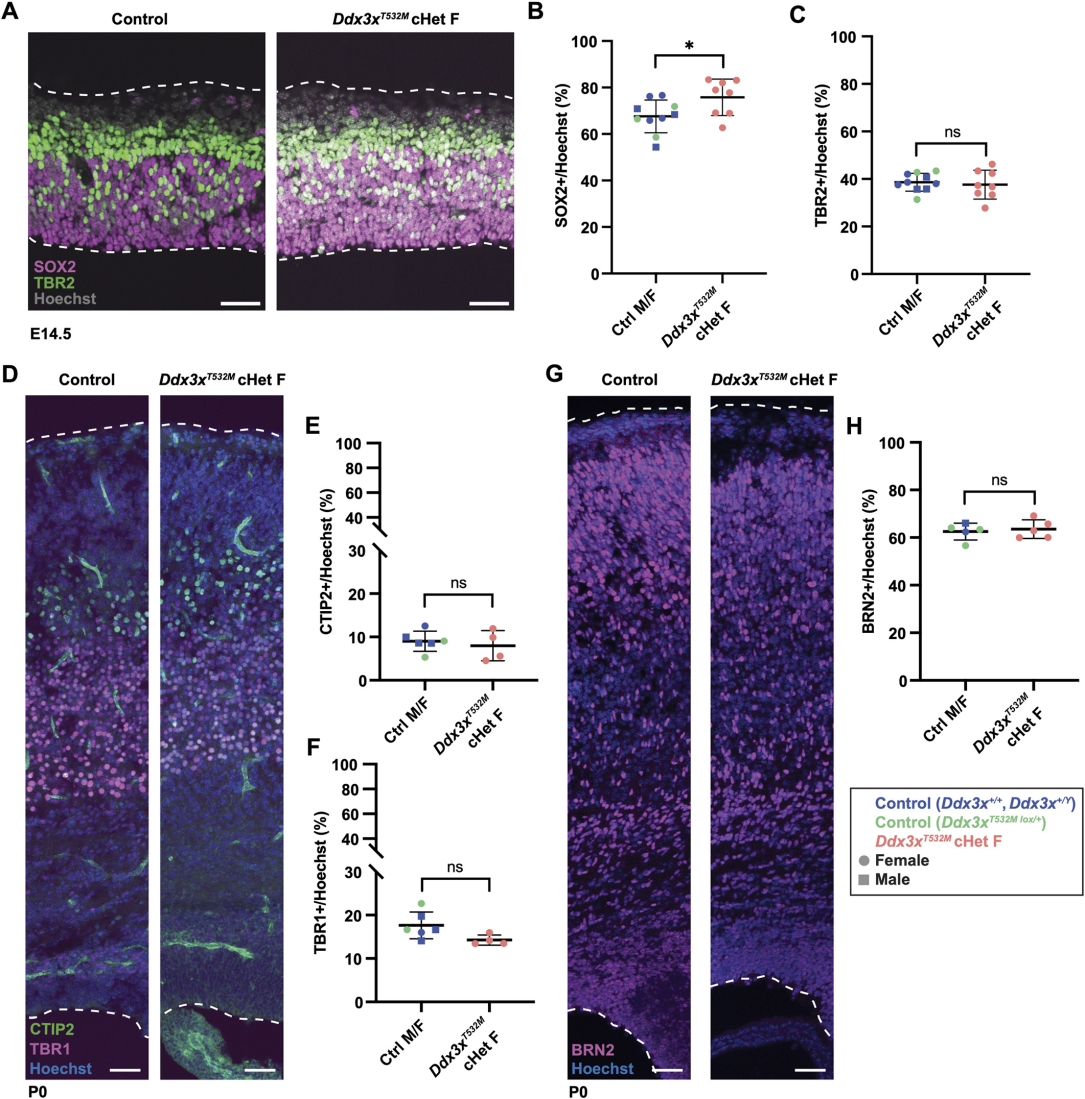
***Ddx3x^T532M^* cHet females have a mild increase in radial glial cells.** (A) Representative images of E14.5 cortices from embryos of denoted genotypes, stained for SOX2 [radial glial cells (RGCs), magenta], TBR2 [intermediate progenitors (IPs), green] and Hoechst (nuclei, gray). White dashed lines outline pial and ventricular surfaces. (B) Quantifications of SOX2^+^ RGC density per total nuclei at E14.5. Squares represent males, and circles represent females; points are color coded by genotype. *n*=10 control males/females, including *n*=7 *Ddx3x^+/+^* or *Ddx3x^+/Y^*, with or without *Emx1*-Cre (blue), and *n*=3 *Ddx3x^T532M lox/+^* females (green); *n*=8 *Emx1*-Cre;*Ddx3x^T532M lox/+^* cHet females (salmon), from seven litters. (C) Quantifications of TBR2^+^ IP density per total nuclei at E14.5. *n*=10 (control males/females, including *n*=7 *Ddx3x^+/+^* or *Ddx3x^+/Y^*, with or without *Emx1*-Cre (blue), and *n*=3 *Ddx3x^T532M lox/+^* females (green); *n*=8 *Emx1*-Cre;*Ddx3x^T532M lox/+^* cHet females (salmon), from seven litters. Immunofluorescence results and quantifications representative of two independent immunofluorescence experiments. (D) Representative images of P0 cortices from embryos of denoted genotypes, stained for TBR1 (layer VI excitatory neurons, magenta), CTIP2 (layer V excitatory neurons, green) and Hoechst (nuclei, blue). White dashed lines outline pial and ventricular surfaces. (E,F) Quantification of neuron density per total nuclei at P0 for TBR1^+^ layer VI (E) and CTIP^+^ layer V (F). Squares represent males, and circles represent females; points are color coded by genotype. *n*=7 (control males/females, including *n*=4 *Ddx3x^+/+^* or *Ddx3x^+/Y^*, with or without *Emx1*-Cre (blue), and *n*=3 *Ddx3x^T532M lox/+^* females (green); *n*=4 *Emx1*-Cre;*Ddx3x^T532M lox/+^* cHet females (salmon), from six litters. (G) Representative images of P0 cortices from embryos of denoted genotypes, stained for BRN2 (layer II/III excitatory neurons, magenta) and Hoechst (nuclei, blue). White dashed lines outline pial and ventricular surfaces. (H) Quantification of BRN2^+^ layer 2/3 neuron density per total nuclei at P0. Squares represent males, and circles represent females; points are color coded by genotype. *n*=5 (control males/females, including *n*=2 *Ddx3x^+/+^* or *Ddx3x^+/Y^*, with or without *Emx1*-Cre (blue), and *n*=3 *Ddx3x^T532M lox/+^* females (green); *n*=5 *Emx1*-Cre;*Ddx3x^T532M lox/+^* cHet females (salmon), from seven litters. Immunofluorescence results and quantifications representative of two independent immunofluorescence experiments. For B,C,E,F,H, each point is the average of two to three quantified coronal sections per single embryo. Points are color coded by genotype as described above*.* Statistics are Student's unpaired, two-tailed *t*-test (B,C,E,F,H). *P<0.05; ns, not significant. Error bars, mean±s.d. Scale bars: 50 µm (A,D,G).

The six known patients with *DDX3X^T532M^* variants are female, as are the vast majority of *DDX3X* syndrome cases. Thus, to understand mechanisms relevant for the patient cohort, we centered our subsequent analyses on *Ddx3x^T532M^* cHet females. We previously showed that *Ddx3x* loss increases progenitor number in the developing cortex (Hoye et al., 2022; Lennox et al., 2020). Thus, we quantified RGCs and IPs in embryonic brains of *Ddx3x^T532M^* cHet females and littermate controls. Relative to controls, including Cre-negative *Ddx3x^T532M lox/+^* female controls, the number of SOX2^+^ RGCs was significantly increased at E14.5, both in total numbers and as a percentage of all cells (Fig. 5A,B; Fig. S3A). However, the number of TBR2^+^ IPs was not significantly different from that in littermate controls (Fig. 5A,C; Fig. S3B). These results demonstrate that *Ddx3x^T532M^* has a mild impact on progenitor composition in cHet females, similar to *Ddx3x^LoF^* in cHet females (Hoye et al., 2022; Lennox et al., 2020).

We next determined the impact of *Ddx3x^T532M^* on the composition of excitatory neurons. We measured the number and density of cortical neurons in *Ddx3x^T532M^* cHet female and control brains at P0, as neurogenesis is complete by this stage (Qian et al., 2000). Quantifications revealed no significant difference in the percentage of deep layer neurons, labeled by either TBR1 (layer VI) or CTIP2 (layer V), or in upper layer BRN2^+^ (layer 2/3) neurons in *Ddx3x^T532M^* cHet female cortices compared to control cortices (Fig. 5D-H). Overall, this subtle effect on neurogenesis is consistent with the mild reduction in brain size of P0 *Ddx3x^T532M^* cHet females*.* It is also similar to *Ddx3x* haploinsufficiency, which causes a subtle impact on excitatory neuron number at P0, with the strongest phenotype evident in *Ddx3x^LoF^* cHemi males (Hoye et al., 2022). Together, these results suggest that, at the tissue and cellular level, *Ddx3x^T532M^* has overall mild impact on cortical neurogenesis in cHet females.

### *In vivo* polysome profiling identifies transcriptional and translational alterations in *Ddx3x^LoF^* cHet and *Ddx3x^T532M^* cHet female cortices

**Fig. 6.**
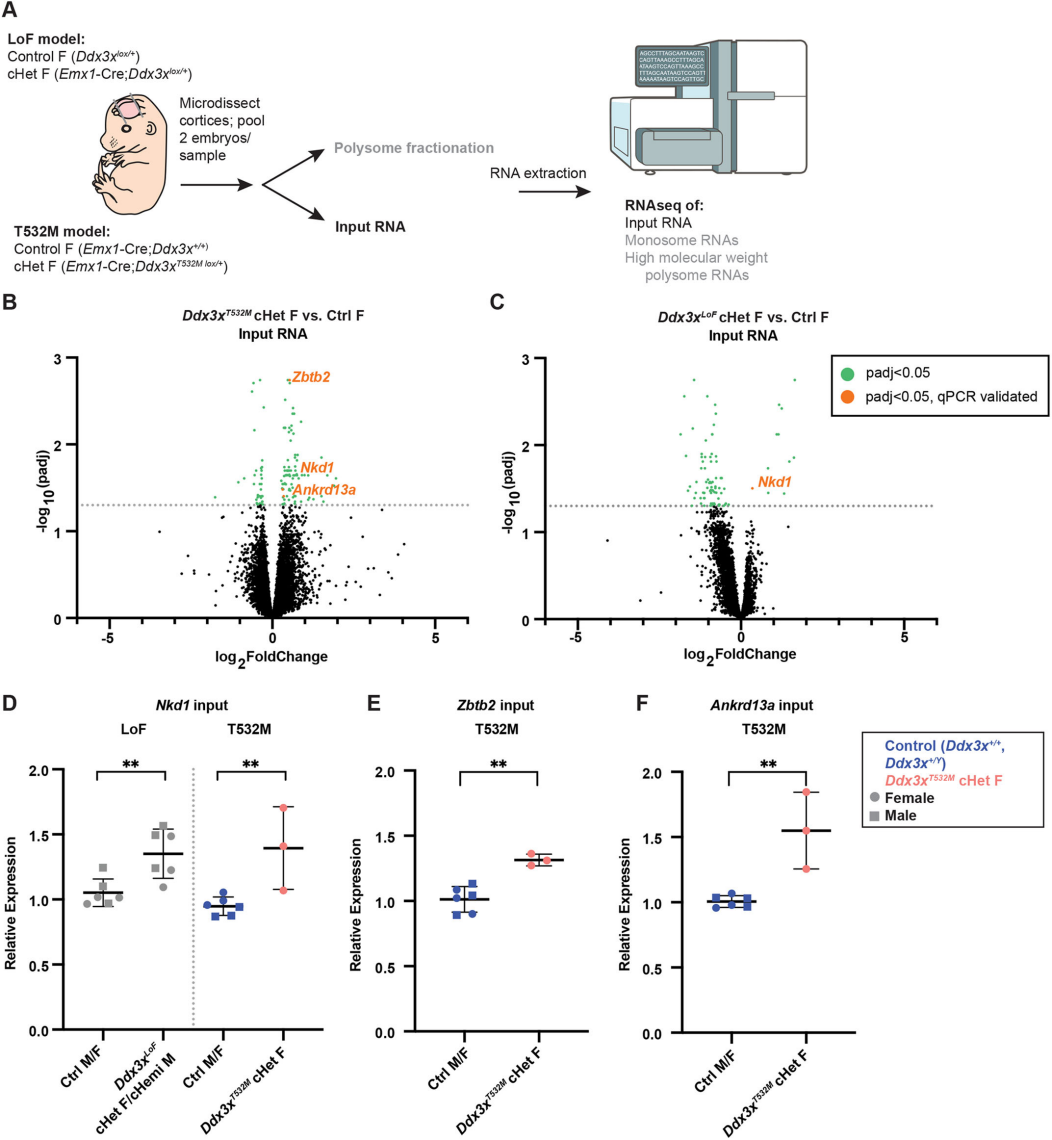
**Comparative RNA sequencing reveals transcriptome differences between *Ddx3x^T532M^* cHet and *Ddx3x^LoF^* cHet female cortices.** (A) Schematic of experimental design for indicated genotypes, including representative polysome fractionation trace annotated with fractions used for RNA isolation. Next-gen sequencer illustration from NIH BioArt Source (see Materials and Methods). (B) Volcano plot showing differentially expressed (DE) transcripts in input RNA sequencing (RNAseq) of *Ddx3x^T532M^* cHet females versus controls. Each point represents a gene; points in green, adjusted *P*-value (padj)<0.05; points in orange, validated via qRT-PCR. Dotted line indicates level of significance (padj<0.05). Note that one gene with a high log_2_ fold change (padj>0.05) is excluded from plot for visualization. (C) Volcano plot showing DE transcripts in input RNAseq of *Ddx3x^LoF^* females versus controls. Each point represents a gene; points in green, padj<0.05; points in orange, padj<0.05 and validated via qRT-PCR. Dotted line indicates level of significance (padj<0.05). (D) qRT-PCR for *Nkd1* in input RNA samples of denoted genotypes. *n*=3 samples/genotype from five litters (T532M); *n*=3 samples/genotype from six litters (LoF). (E) qRT-PCR for *Zbtb2* in input RNA samples of denoted genotypes. *n*=3 samples/genotype from five litters. (F) qRT-PCR for *Ankrd13a* in total RNA samples of denoted genotypes. *n*=3 samples/genotype from five litters. For D-F, each point represents a single sample (from pooled pair of cortices of like genotype), assayed in technical triplicate. For D-F, squares represent males, and circles represent females; points are color coded by genotype. All qRT-PCR results are first normalized to β-actin, then to control females of the appropriate model. qPCRs were performed once. Statistics are Student's unpaired, two-tailed *t*-test (D-F). **P<0.005. Error bars, mean±s.d.

We next aimed to understand the molecular basis by which *Ddx3x^T532M^* impacts neurogenesis. Previous ribosome profiling studies showed that E11.5 *Emx1*-Cre;*Ddx3x^lox/lox^* [*Ddx3x^LoF^* conditional homozygous (cHomo)] female and *Ddx3x^LoF^* cHemi male cortices have decreased translational efficiency of a small number of transcripts, with mild effects on mRNA transcript levels (Hoye et al., 2022). In addition, *DDX3X^T532M^* expression can impact transcript abundance in primary cortical progenitors (Mosti et al., 2025). Thus, we postulated that *Ddx3x^T532M^* cHet female cortices also exhibit altered mRNA levels. Given the severe impact of *Ddx3x^T532M^* on RNA duplex unwinding (Lennox et al., 2020), we further predicted that *Ddx3x^T532M^* and *Ddx3x^LoF^* have qualitative differences in translation. Thus, to globally assess RNA abundance and translational regulation, we performed polysome profiling in cortices from both the *Ddx3x^T532M^* cHet and *Ddx3x^LoF^* cHet female mouse models, in addition to those from their littermate controls (Fig. 6A). We performed this analysis at E14.5, as both models exhibit progenitor phenotypes at this stage.

As confirmation of the RNA-sequencing (RNAseq) strategy, we first assessed *Ddx3x* in input samples from control and cHet females of both genotypes. Compared to control females, *Ddx3x^LoF^* cHet females had more reads aligning with exons 1-3 and a drop-off of reads aligning with exons 4-17 (Fig. S4A). This is consistent with the design of this floxed model, which employs loxP sites flanking exons 4-17 (Chen et al., 2016a). Additionally, when considering only reads that align to the targeted *Ddx3x* exon 14, *Ddx3x^T532M^* cHet females express both *Ddx3x^WT^* and *Ddx3x^T532M^* alleles at the expected 1:1 ratio (Fig. S4B). Taken together, these analyses confirm that both models behave as expected and support further exploration of this dataset.

We next sought to understand the global impact of *Ddx3x^T532M^* and *Ddx3x^LoF^* on total transcript abundance. *Ddx3x^T532M^* cHet female cortices had 121 differentially expressed (DE) transcripts compared to their controls, including 86 upregulated and 35 downregulated transcripts [adjusted *P*-value (padj)<0.05; Fig. 6B; Table S1]. In contrast, 86 total DE transcripts were identified in *Ddx3x^LoF^* cHet female cortices, with 11 upregulated and 75 downregulated (padj<0.05, Fig. 6C; Table S2). There was very little qualitative overlap in transcriptomic changes, with only two shared DE genes among the two models. Gene ontology (GO) analysis showed that *Ddx3x^T532M^* affects transcripts associated with translation (padj<0.05; Fig. S4C, Table S1), whereas *Ddx3x^LoF^* affects transcripts associated with DNA binding and transcription (padj<0.05, Fig. S4D, Table S2). Notably, the magnitude of these transcriptional changes was mild (< 2-fold) for both genotypes (Fig. 6B,C). This is consistent with both the major role of DDX3X in translation regulation and with our previous data from E11.5 *Ddx3x^LoF^* cHomo female and *Ddx3x^LoF^* cHemi male cortices (Hoye et al., 2022).

We next validated transcriptional changes. Only one transcript, *Nkd1*, was increased in expression in both cHet models compared to their controls (Fig. 6B,C). *Nkd1* encodes a negative regulator of Wnt signaling, a pathway affected by *DDX3X* (Yang et al., 2019; Dolde et al., 2018; Cruciat et al., 2013; Snijders Blok et al., 2015; Lennox et al., 2020). *Nkd1* differential expression was validated in *Ddx3x^LoF^* cHet and *Ddx3x^T532M^* cHet female cortices via qPCR (Fig. 6D). We also validated transcripts with expression differences specific to *Ddx3x^T532M^* cHet females. *Zbtb2*, a transcription factor involved in cell state transitions (Olivieri et al., 2021), and *Ankrd13a*, a ubiquitination-sensing protein involved in cell death checkpointing (Won et al., 2022), were both significantly increased in expression in *Ddx3x^T532M^* cHet females versus controls by both RNAseq and qRT-PCR (Fig. 6E,F). Taken together, these findings indicate that both *Ddx3x^T532M^* and *Ddx3x^LoF^* have mild impacts on RNA abundance, consistent with the predominant function of DDX3X as a translational regulator. Additionally, the DE genes within each genotype are almost entirely distinct, suggesting different transcriptional responses to *Ddx3x^T532M^* and *Ddx3x^LoF^* alleles.

Next, we asked whether similar molecular changes are observed in *Ddx3x^T532M^* cHemi male mice, which have more severe brain phenotypes than those of their female counterparts (Figs 3 and 4). To address this, we employed FACS isolation of Cre-positive live cells labeled via *Rosa26^Ai14^* in E11.5 cortices of all genotypes across both conditional models (Fig. S5A). This stage allowed us to retrieve live cells from the cortices of *Ddx3x^T532M^* cHemi male and *Ddx3x^LoF^* cHomo female mice, which both exhibit notable apoptosis as development proceeds (Fig. 4C) (Hoye et al., 2022). We then performed qPCR analysis of *Ankrd13a*, *Nkd1* and *Zbtb2*, the three targets validated at E14.5, as well as *Rcor2*, a previously identified translational target of DDX3X at E11.5 (Hoye et al., 2022). Consistent with our previous results, both *Ddx3x^LoF^* cHemi males and *Ddx3x^LoF^* cHomo females demonstrated slight ∼1.2-fold upregulation of *Rcor2* compared to respective littermate controls, while the other targets were unchanged (Fig. S5B-E). In contrast, *Ddx3x^T532M^* cHemi male cortices exhibited significant 4- to 6-fold increases in expression of all four transcripts compared to male and female control cortices and to *Ddx3x^T532M^* cHet female cortices (Fig. S5F-I). Altogether, these data suggest there are distinct quantitative and qualitative differences in molecular targets for *Ddx3x^T532M^* cHemi males compared to *Ddx3x^T532M^* cHet females and to *Ddx3x^LoF^* cHemi males. These differences are consistent with the more pronounced microcephaly seen in *Ddx3x^T532M^* cHemi males relative to other genotypes.

**Fig. 7.**
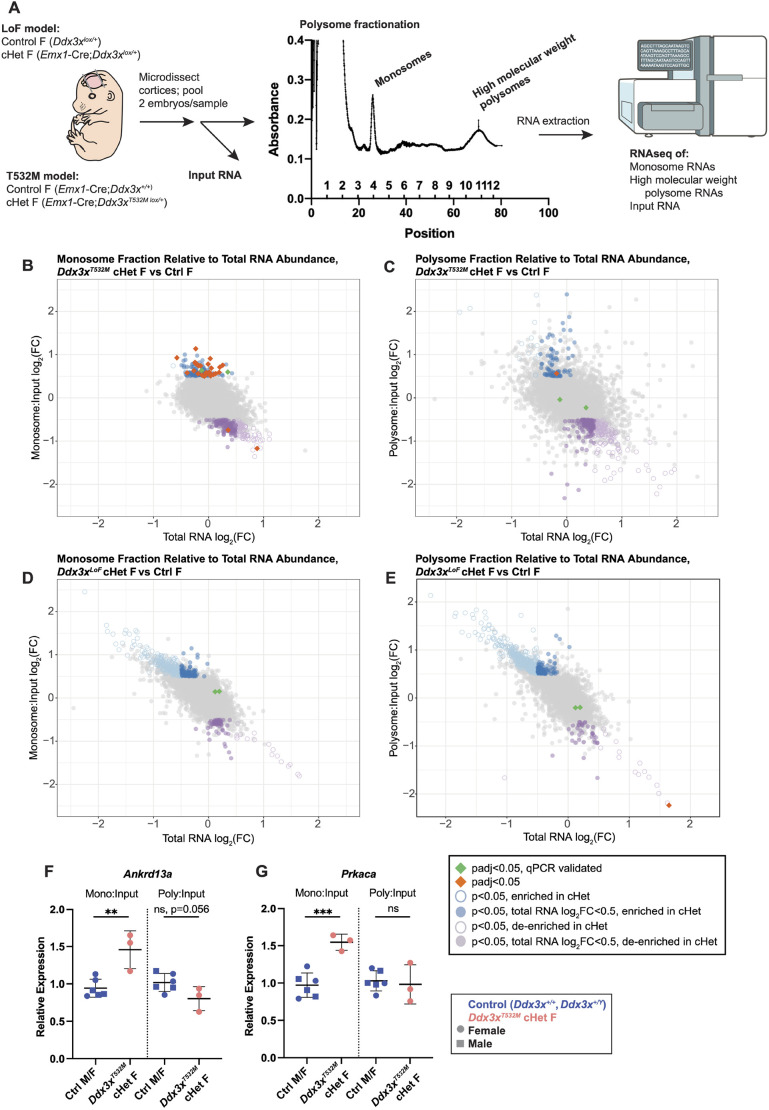
**Comparative polysome profiling reveals differences between *Ddx3x^T532M^* cHet and *Ddx3x^LoF^* cHet female cortices.** (A) Schematic of experimental design, for indicated genotypes, including representative polysome fractionation trace annotated with fractions collected for RNA isolation. Next-gen sequencer illustration from NIH BioArt Source (see Materials and Methods). (B) Plot showing transcripts enriched in monosome-associated fraction over input in RNAseq of *Ddx3x^T532M^* cHet females versus controls. FC, fold change. (C) Plot showing transcripts enriched in polysome-associated fraction over input in RNAseq of *Ddx3x^T532M^* cHet females versus controls. (D) Plot showing transcripts enriched in monosome-associated fractions over input in RNAseq of *Ddx3x^LoF^* cHet females versus controls. (E) Plot showing transcripts enriched in polysome-associated fraction over input in RNAseq of *Ddx3x^LoF^* cHet females versus controls. (F) qRT-PCR for *Ankrd13a* in monosome/polysome fractions of denoted genotypes, normalized for input. *n*=3 samples/genotype from five litters. (G) qRT-PCR for *Prkaca* in monosome/polysome fractions of denoted genotypes, normalized for input. *n*=3 samples/genotype from five litters. All qRT-PCR results are first normalized to β-actin, then to control females. Each point represents a single sample (coming from cortices of a pooled pair of like-genotype embryos, as in A), assayed in technical triplicate. For F-G, squares represent males, and circles represent females; points are color coded by genotype. qPCRs were performed once. Statistics are Student's unpaired, two-tailed *t*-test (F-G). **P<0.005; ***P<0.0005; ns, not significant. Error bars, mean±s.d.

Next, we sought to assess translational regulation in *Ddx3x^T532M^* cHet and *Ddx3x^LoF^* cHet female cortices. We measured mRNAs found in high-molecular-weight polysome and monosome fractions (Fig. 7A), as mRNAs associated with more ribosomes are inferred to be more translationally active than those associated with fewer ribosomes (Arava et al., 2003). Given its role in translational initiation and prior data from *Ddx3x^LoF^* cHemi male and cHomo female cortices (Hoye et al., 2022), we expected that DDX3X translational targets may shift from polysome-associated fractions to monosome-associated fractions. To assess this, we evaluated enrichment of protein-coding RNAs in either monosome- or polysome-associated fractions, relative to their input RNA abundance. We identified 51 transcripts significantly altered in monosomes and only one in polysomes of *Ddx3x^T532M^* cHet females compared to controls (padj<0.05; Fig. 7B,C; Tables S3 and S4). Of the 51 significant monosome-associated transcripts, 48 were increased, consistent with our expectation that *Ddx3x^T532M^* expression would impact translation initiation. Additionally, GO analysis showed that monosome-associated transcripts included numerous RNA regulatory factors such as ribosome-associated proteins (padj<0.05, Fig. S5J, Table S3). In contrast, only one transcript was significantly altered in polysomes, with none significantly altered in monosomes, from *Ddx3x^LoF^* cHet female cortices (padj<0.05; Fig. 7D,E; Tables S5 and S6). There was no overlap in targets identified between these genotypes, consistent with the transcriptomic datasets.

We next validated translational targets affected by *Ddx3x^T532M^* expression via qRT-PCR analysis. We selected two candidates, *Prkaca* and *Ankrd13a*, which have previously been shown to have decreased translational efficiency in response to *DDX3X* depletion in cell culture (Calviello et al., 2021). *Prkaca* is the catalytic subunit of protein kinase A, which is ubiquitously expressed and critical for development (Skålhegg et al., 2002; Turnham and Scott, 2016; Huang et al., 2002). *Prkaca* and *Ankrd13a* were both significantly enriched in monosome-associated fractions via RNAseq in *Ddx3x^T532M^* cHet females (Fig. 7B, green diamonds). Whereas *Ankrd13a* is also transcriptionally upregulated in *Ddx3x^T532M^* cHet females (Fig. 6E), *Prkaca* is not DE at the total RNA level (Fig. S5K). As expected, qPCR results showed similar significant changes in *Ddx3x^T532M^* cHet females compared to controls, with both *Ankrd13a* and *Prkaca* transcripts over-represented in monosome-associated fractions (Fig. 7F,G).

In total, these profiling experiments demonstrate the critical role of DDX3X in translational regulation during corticogenesis. These data show that, in heterozygous female cortices, *Ddx3x^T532M^* and *Ddx3x^LoF^* both impact RNA abundance at E14.5, but with notable qualitative differences, while only *Ddx3x^T532M^* has an appreciable effect on translational regulation. Taken together, these data for both steady-state RNA abundance and translational regulation indicate that *Ddx3x^T532M^* and *Ddx3x* haploinsufficiency in the cortex have distinct impacts on gene expression.

## DISCUSSION

This work describes the first conditional knock-in model for a *DDX3X* syndrome missense variant. Heterozygous *de novo* variants in *DDX3X* cause *DDX3X* syndrome and are associated with both monogenic intellectual disability and autism. Half of the ∼200 mutations reported to date are missense, a subset of which have been classified as severe based on clinical and biochemical findings (Lennox et al., 2020; Mosti et al., 2025). Here, to understand the cellular and molecular mechanisms by which these clinically severe variants affect cortical development, we developed and leveraged a new genetic mouse model of the missense variant *Ddx3x^T532M^*. We discover that *Ddx3x^T532M^* has overall mild impacts on neurogenesis and define its molecular targets *in vivo*. Our investigations illustrate the value of the *Ddx3x^T532M^* mouse model for understanding DDX3X biology and disease.

We carefully characterized the *Emx1*-Cre;*Ddx3x^T532M lox/+^* (*Ddx3x^T532M^* cHet) female model for *Ddx3x* RNA and protein expression. Importantly, using an allelic discrimination qRT-PCR assay, as well as monitoring association with polysomes and monosomes, we show that both alleles are equivalently expressed in *Ddx3x^T532M^* cHet female embryonic brains. Thus, this mouse model faithfully recapitulates heterozygous mutations associated with *DDX3X* syndrome. Unexpectedly, we quantified mild overexpression of total *Ddx3x* RNA and protein in *Ddx3x^T532M^* cHet cortices. Notably, lentiviral-based expression of *DDX3X^WT^* or missense variants in primary mouse cortical progenitors also induces a slight overexpression of total DDX3X protein without any phenotypic impact (Mosti et al., 2025). Given this, we favor that slightly elevated levels of DDX3X are unlikely to drive the phenotypes observed in this mouse model. Future studies will be needed to determine the extent to which DDX3X levels in this mouse model influence phenotypes in the brain and other tissues. As *DDX3X* RNA and protein levels have yet to be characterized in *DDX3X* syndrome, the clinical relevance of this mild overexpression is unknown.

### *Ddx3x^T532M^* and *Ddx3x^LoF^* mouse models have sexual dimorphic impacts on corticogenesis

At a tissue and cellular level, *Ddx3x^T532M^* cHet females largely phenocopy *Ddx3x^LoF^* cHet females (Hoye et al., 2022). *Ddx3x^T532M^* cHet females showed a very mild decrease in overall cortex size at P0, while *Ddx3x^LoF^* cHet females are phenotypically indistinguishable from controls. Embryonically, *Ddx3x^T532M^* cHet and *Ddx3x^LoF^* cHet females both contain slightly more RGCs, and postnatally both models trend towards having fewer neurons. While neuron number is only significantly altered in *Ddx3x^LoF^* cHet cortices, it is notable that previous analyses included both *Ddx3x^LoF^* cHemi males and *Ddx3x^LoF^* cHet females, with the former being more phenotypic (Hoye et al., 2022). Overall, the *Ddx3x^T532M^* cHet and *Ddx3x^LoF^* cHet female models exhibit largely similar mild cellular and tissue-level outcomes in the cortex.

Compared to *Ddx3x^T532M^* cHet females, *Ddx3x^T532M^* cHemi males have severe microcephaly and apoptosis. Similarly, *Ddx3x^LoF^* cHemi males trended toward stronger cellular and molecular phenotypes than their haploinsufficient female counterparts (Hoye et al., 2022). Consistent with this, germline *Ddx3x* loss is embryonic lethal in *Ddx3x^−/Y^* male mice, but not in heterozygous *Ddx3x^+/−^* females (Chen et al., 2016a; Boitnott et al., 2021). This has been suggested as an explanation for why there are fewer affected males with *DDX3X* syndrome. However, the cause of the observed sexual dimorphism in *Ddx3x^T532M^* cHemi male mice remains unclear. We speculate that this could be due to lack of compensation by *Ddx3y*; however, both *Ddx3y* and *Ddx3x* are upregulated in *Ddx3x^T532M^* cHemi male cortices. Additional analyses before the onset of apoptotic cell death will be valuable to better understand the molecular underpinnings of this microcephaly phenotype observed in *Ddx3x^T532M^* cHemi male mice.

Our *in vivo* analysis of *Ddx3x^T532M^* expands on cellular phenotypes observed *in vitro DDX3X^T532M^* studies. Similar to the *in vivo* findings, acute induction of *DDX3X^T532M^* in primary mouse cortical progenitors shifts cell fate decisions toward progenitor self-renewal (Mosti et al., 2025). However, unlike *Ddx3x^T532M^* cHet female brains, these *in vitro* models also show increased neuronal death. This difference may result from a mixed male/female cell population used for lentiviral transduction or the acute induction used in this *in vitro* system. Taken together, our recent studies highlight the importance of using diverse approaches to understand the molecular mechanisms of *DDX3X* syndrome.

### *Ddx3x^T532M^* cHet and *Ddx3x^LoF^* cHet female cortices have distinct transcriptomic and translational changes

In our molecular analyses of translation and RNA abundance in the cortex, we observed notable differences between *Ddx3x^T532M^* cHet and *Ddx3x^LoF^* cHet females. *Ddx3x^T532M^* cHet female cortices had a small, but significant, number of transcripts with altered translation patterns. In contrast, translation was not impacted in *Ddx3x^LoF^* cHet female cortices, which had only one transcript significantly under-represented in polysome-associated fractions. At the transcriptome level, each model's DE genes were also qualitatively distinct. *Ddx3x^T532M^* cHet females showed enrichment of ribosome-associated mRNAs; in *Ddx3x^LoF^* cHet females, expression of transcription factors was largely affected. Additionally, some targets in *Ddx3x^T532M^* cHet females exhibited over-representation in monosome fractions as well as increases in mRNA expression, as exemplified by *Ankrd13a.* This suggests a potential compensatory gene expression mechanism, also seen in E11.5 *Ddx3x^LoF^* cHomo female and cHemi male brains (Hoye et al., 2022) and in a *DDX3X* degron model (Jowhar et al., 2024). Overall, these molecular profiling experiments suggest that, despite its mild cellular phenotypes, *Ddx3x^T532M^* may not strictly behave as a LoF variant in cHet females. Future work, including functional investigation of DDX3X translational targets, will help inform how *Ddx3x^T532M^* and *Ddx3x^LoF^* differentially impact brain development. These molecular analyses are consistent with the canonical role of DDX3X in translation initiation for a subset of transcripts (Calviello et al., 2021; Chen et al., 2018; Phung et al., 2019). Interestingly, *Ddx3x^T532M^* cHet females showed enrichment of translation machinery-associated mRNAs in both the transcriptome and in monosome-associated fractions, suggesting that DDX3X^T532M^ could influence translation both directly and indirectly. This aligns with recent proteomic studies showing that translation-regulating proteins are decreased in *Ddx3x* haploinsufficient murine neurons (Mossa et al., 2025).

How might DDX3X^T532M^ and DDX3X^LoF^ uniquely control translation? DDX3X^T532M^ is helicase dead (Lennox et al., 2020), and failure to unwind RNA secondary structures in 5′ UTRs is known to cause accumulation of monosomes at upstream open reading frames (uORFs) (Iwasaki et al., 2016; Heyer and Moore, 2016). This may allow translation machinery to promiscuously use uORFs upstream of the standard translational start site. Thus, *Ddx3x^T532M^* cHet female monosome fractions may contain transcripts derived from uORF translation, whereas *Ddx3x^LoF^* cHet female monosome fractions may not have the same bias. Future studies will be needed to investigate these mechanisms and tease apart these possibilities.

### Key questions in understanding *DDX3X* syndrome

It is notable that the *Ddx3x^T532M^* cHet model had relatively mild phenotypes. Based on its associated clinical and molecular phenotypic severity, including a lack of *in vitro* helicase function (Mosti et al., 2025; Lennox et al., 2020), we expected to observe more severe phenotypes in *Ddx3x^T532M^* cHet females than in *Ddx3x^LoF^* cHet females. Of note, although the four individuals initially identified with this variant presented with concordant severe neurodevelopmental deficits, one individual carrying the *DDX3X^T532M^* variant has since been identified and presents with milder clinical findings, notably lacking PMG (Lennox et al., 2020; Dai et al., 2022). Thus, it may be that *DDX3X^T532M^* variants cause a wider spectrum of clinical presentations, which span from mild to severe, than was previously appreciated. As more molecular lesions are identified in *DDX3X*, we will learn more about the clinical heterogeneity of *DDX3X* syndrome. In addition, skewed X inactivation favoring the wild-type allele has been observed in lymphocytes from individuals with *DDX3X* syndrome, with some demonstrating near complete inactivation of the pathogenic allele (Snijders Blok et al., 2015). Differences in X inactivation between mice and humans may also underlie some of the divergent phenotypes we have observed in our model.

Cortex-specific *Ddx3x^T532M^* expression can model some, but not all, aspects of *DDX3X* syndrome, in which *de novo* mutations occur early in development and affect all tissues. For example, individuals with *DDX3X^T532M^* mutations also present with congenital cardiac defects (Lennox et al., 2020). Thus, using this model with additional germline and tissue-specific Cre drivers will be important to fully understand the *in vivo* impacts of this variant. Additionally, postnatal analysis into the effects of *Ddx3x^T532M^* on neural circuitry and behavior would be particularly valuable, as phenotypes in these areas have been characterized in *Ddx3x^LoF^* models (Boitnott et al., 2021; Mossa et al., 2025). Further, future studies may use this model to assess additional molecular functions of DDX3X, such as regulating mRNA stability (Jowhar et al., 2024).

This study establishes a new mouse model for investigation of *DDX3X^T532M^* physiological, cellular and molecular impacts. By generating a mouse model representative of one recurrent severe *DDX3X* syndrome variant, we establish a paradigm in which to understand phenotypes associated with other *DDX3X* missense mutations. Future investigations into the breadth of missense *DDX3X* variants will be invaluable to better understand underlying mechanisms of *DDX3X* syndrome and related disorders.

## MATERIALS AND METHODS

### Mouse husbandry

All animal use was approved by the Duke Division of Laboratory Animal Research and Institutional Animal Care and Use Committee. Mouse strains used are as follows and are genotyped as previously described: *Emx1-*Cre (005628, The Jackson Laboratory) (Gorski et al., 2002); *Rosa26^Ai14^* (007914, The Jackson Laboratory) (Madisen et al., 2010); *Ddx3x^lox^* (gift from Li-Ru You; Chen et al., 2016a); *ROSA26*::FLPe knock-in (009086, The Jackson Laboratory) (Farley et al., 2000); *Ddx3x^T532M lox^* (this paper, detailed below). Embryonic time points are defined as E0.5 being the date of plug identification. Postnatal timepoints are defined as date of birth being P0. For the *Ddx3x^T532M lox^* model, controls used include (1) *Ddx3x^+/+^* males and females; (2) *Emx1*-Cre;*Ddx3x^+/+^* males and females; and (3) *Ddx3x^T532M lox/+^* females. For the *Ddx3x^lox^* model, controls used include (1) *Emx1*-Cre;*Ddx3x^+/+^* males and females; and (2) *Ddx3x^lox/+^* females and *Ddx3x^lox/Y^* males*.* Specific details about controls for each experiment can be found in figure legends.

### Statistical methods and rigor

Description of specific *n*, statistical tests and *P*-values for each experiment are reported in the figure legends. For all experiments, male and female mice were used as designated, and embryo sex was determined via *Sry* genotyping. When possible, control littermates were used for comparison. All analyses were performed by investigators unaware of genotype. All non-RNAseq statistical analyses were performed using GraphPad Prism (8.4.3).

### Conditional knock-in mouse generation

Founder mice chimeric for the *Ddx3x^T532M lox^* allele were generated by the Duke Transgenic Mouse Shared Resource using standard gene-targeting techniques in 129/B6N hybrid mouse ES cells to introduce into the endogenous intron 11-12 of *Mus musculus* (*Mm*) *Ddx3x* a minigene containing the following: an upstream loxP site, cDNA for wild-type *MmDdx3x* exons 12-17, followed by a bovine growth hormone polyA signal, a neomycin selection cassette itself flanked by *frt* sites, and then a downstream loxP site. ES cells were also targeted to introduce the T532M mutation (ACA>ATG) into the endogenous exon 14 of *MmDdx3x*. After screening mosaic founders for germline transmission, carriers were bred to *Rosa26*::FLPe knock-in mice (Farley et al., 2000) to excise the neomycin selection cassette; this final construct is depicted in Fig. 1B. *Ddx3x^T532M lox^* mice were genotyped using the following primers: T532M-S2-F (5′-CACTATGCCTCCAAAAGGTGTCCG-3′) and oAJP8-R (5′-GTTCTCTAACCAAGAAGGCAC-3′), resulting in a ∼1500 bp band from a *Ddx3x^WT^* allele and an ∼800 bp band from a *Ddx3x^T532M lox^* allele (see Fig. 1D). Mice for the included experiments are from F3 or later generations backcrossed to C57BL/6J.

### FACS and RNA extraction

Samples were processed for FACS as previously described (Alsina et al., 2024) and sorted at 6°C using either a Beckman-Coulter Astrios or Sony SH8000 cell sorter. Singlets were gated using side scatter and forward scatter, and live, Cre-positive cells were selected using gates for DAPI^−^ and tdTomato^+^ populations, respectively. Cells were sorted into TRIzol LS Reagent (Invitrogen, 10296028), and RNA was extracted per the manufacturer's protocol using either TRIzol LS reagent or the Zymo Direct-zol RNA Miniprep kit (Zymo Research, R2050).

### Cell culture

HEK-293T immortalized cells (ATCC, CRL-3216) were maintained at 37°C, 5% CO_2_ in DMEM high-glucose medium (Gibco, 11965092) supplemented with 10% fetal bovine serum (HyClone, SH30088.03HI) and 1% Pen-Strep (Gibco, 15140122).

### qRT-PCR analysis

qRT-PCR analyses of gene expression were performed as previously described (Hoye et al., 2022), using iScript reverse transcriptase (Bio-Rad, 1708891) for cDNA synthesis per the manufacturer's protocols. iTaq University SYBR Green Supermix (Bio-Rad, 1725124) was used to prepare qRT-PCR reactions, along with primers for specific genes of interest. Primers for *Ddx3x*, *Ddx3y*, *Rcor2* and β-actin are as previously published (Hoye et al., 2022). The remaining primer sequences were sourced from PrimerBank (Wang and Seed, 2003; Spandidos et al., 2008, 2010): *Ankrd13a* (29165672a1), *Nkd1* (31980622a1), *Prkaca* (7110693a1), *Zbtb2* (226423892c1). All qRT-PCRs were run on either a QuantStudio 3 or QuantStudio 6 Pro Real-Time PCR system (Applied Biosystems). Relative expression was calculated by normalizing genes of interest to β-actin, then to the average of controls.

#### Allelic discrimination

For allelic discrimination, a Thermo Fisher Scientific Custom TaqMan Gene Expression Assay (4332077, assay ID ANDKDFZ) was developed to discriminate between *MmDdx3x^WT^* (probe conjugated to VIC fluorophore) and *MmDdx3x^T532M^* (probe conjugated to FAM fluorophore). To validate this assay, we generated various titration controls using HEK-293T cells transfected with various ratios of plasmids containing GFP-tagged *MmDdx3x^WT^* and/or *MmDdx3x^T532M^* under the CAG promoter. Mock HEK-293T transfection controls were included to ensure specificity of this allelic TaqMan assay for mouse cDNA versus human cDNA. Importantly, this TaqMan assay does not amplify the endogenous human *DDX3X* expressed in HEK-293T cells (gray traces present as short lines at the data's origin point in Fig. S1A,C,F). TaqMan RT-qPCR assays were set up using the TaqMan Gene Expression Master Mix (Thermo Fisher Scientific, 4369016). All qRT-PCRs were run on either a QuantStudio 3 or QuantStudio 6 Pro Real-Time PCR system (Applied Biosystems). For assessment in Fig. 2B,G and Fig. S1B, the endpoint fluorescence values of FAM and VIC for each sample were used to calculate the FAM/VIC (or *MmDdx3x^T532M^/MmDdx3x^WT^*) ratio, which was averaged across technical triplicates, then normalized to the 50:50 titration control. For ease of visualization of these exponential data, points in Fig. 2B,G and Fig. S1B are transformed as follows: log_10_(normalized *MmDdx3x^T532M^/MmDdx3x^WT^* ratio+1). In Fig. S1A,C,F, embryonic samples are plotted at their average endpoint values for FAM and VIC, and titration controls are plotted as best-fit lines of the average values across all 40 PCR cycles for each independent transfection for ease of comparing to embryonic samples.

#### Polysome fractionation qRT-PCR

For polysome fractionation cDNA synthesis, the extension step of the iScript protocol was increased to 40 min. qRT-PCRs were performed with SYBR Green Supermix as above. Gene expression across different fractions was calculated by first normalizing to β-actin and then normalized to the corresponding expression from input.

### Immunofluorescence

Embryonic and postnatal mouse brains were dissected, fixed and sectioned as previously described (Mao et al., 2015). 20 µm coronal cryosections from the cortex were collected and stored at −80°C until staining. Sections were permeabilized for 20 min at room temperature in 0.25% Triton X-100 in PBS, then blocked in 5% normal goat serum in PBS for 1 h at room temperature. Sections were incubated in primary antibodies diluted in block solution overnight at 4°C, then incubated in appropriate secondary antibodies diluted in PBS for 2 h at room temperature. The following primary antibodies were used: rat anti-SOX2 (Thermo Fisher Scientific, 14-9811-82, RRID:AB_11219471; 1:1000), rabbit anti-TBR2 (Abcam, ab23345, RRID:AB_778267, 1:500), rabbit anti-TBR1 (Cell Signaling Technology, 49661S, 1:1000), mouse anti-CTIP2 (Abcam, ab18465, 1:500), rabbit anti-BRN2 (Novus, NBP2-21585, 1:400) and rabbit anti-CC3 (Cell Signaling Technology, 9661, RRID:AB_2341188, 1:250). Secondary antibodies used were AlexaFluor conjugated, donkey anti-rat/rabbit/mouse as appropriate for primary antibodies used (Thermo Fisher Scientific, 1:500). Following immunostaining, slides were mounted in Vectashield (Vector Labs, H-1000-10).

### Imaging and analysis

Whole-brain images were taken with a Leica M165-FC dissecting scope, using the same zoom settings for brains of the same embryonic stage; from these images, cortical area was quantified via manual tracing of the outline of each cortical hemisphere in Fiji (2.14.0/1.54f) (Schneider et al., 2012), averaging the two measurements, and then normalizing to the average of littermate control measurements. Immunofluorescent images were captured at 5×, 20×, 40× and/or 63× using a Zeiss AxioObserver Z.1 equipped with an Apotome for optical sectioning. For each experiment, three sections per embryo were imaged and quantified. Identical exposures, Z intervals and Apotome phases were used within each experiment. All images within an experiment were post-processed in Fiji/ImageJ to identically adjust brightness/contrast and crop images for subsequent quantifications, which were performed either in Fiji or in QuPath (0.2.3) (Bankhead et al., 2017). Cell counting was performed either manually (Fiji Cell Counter plug-in or QuPath points tool) or automatically (QuPath cell detection tool). The following settings were used for QuPath automatic cell detection: requested pixel size, 0.1 µm; background radius, 5 µm; minimum area, 10 µm^2^; maximum area, 200 µm^2^; cell expansion, 2 µm; include cell nucleus; and smooth boundaries unchecked. The threshold was set for each individual channel, but equivalently across all sections within an experiment (generally between 25 and 150).

### Polysome fractionation

Polysome fractionation was performed as previously described (Hoye et al., 2022). Embryonic cortices were microdissected, flash frozen in liquid nitrogen and stored at −80°C until use. Cortices from two embryos of like genotype were pooled per sample. Cortices were thawed on ice, lysed in 400 µl polysome buffer using a hand blender, then triturated 10× with a 10-gauge needle. Triton X-100 was added to 1%, then lysates were clarified by spinning at 20,000 ***g*** for 10 min at 4°C. 50 µl of clarified lysate was removed as input, to which 150 µl of TRIzol LS was added before storage at −80°C. Clarified lysates were loaded onto 15-50% sucrose gradients prepared in polysome buffer and ultracentrifuged in an SW41 Ti rotor at 35,000 ***g*** for 2 h at 4°C. Gradients were fractionated into 12∼1 ml fractions using a BioComp Piston Gradient Fractionator instrument with a TRIAX flow cell to measure absorbance at 260 nm. RNA was extracted from pooled fractions and input samples using the Zymo Direct-zol RNA Miniprep kit (Zymo Research, R2050).

### Sequencing and bioinformatic analysis

Library preparation and sequencing were performed by the Duke Sequencing and Genomic Technologies Core Facility. RNAseq libraries were prepared for each fraction and the input using the RNAseq library with KAPA HyperPrep kit and 50 bp paired-end reads were sequenced on the NovaSeq X Plus. An average of 55 million reads were generated for each sample. We only considered reads with 95–97% of the bases that were called with >99.9% accuracy. Reads were quality checked using FastQC (v0.11.7) and aligned to the mouse genome (Grm38) using STAR Aligner (2.7.11a).

Raw gene counts were determined using featureCounts (subread/2.0.3). Differential expression of protein coding genes with a minimum read count of ten in one or more of the fractions were compared within each mouse line (T532M or LoF) using DESeq2 (1.34.0) (Love et al., 2014). Changes in RNA abundance (differential expression in the input fraction) as well as fraction enrichment (monosome:input and polysome:input) were recovered for downstream comparisons. Differential gene expression in the input fractions representing changes in total RNA abundance were plotted as a volcano plot using GraphPad Prism. Changes in transcript enrichment in the monosome and polysome fraction (log_2_FoldChange) were plotted against the changes in total RNA abundance (log_2_FoldChange) in R (4.1.3) using ggplot2 (3.5.1). GO analyses were performed by assessing gene lists using the National Institutes of Health (NIH)'s DAVID Bioinformatics resource (Sherman et al., 2022; Huang et al., 2009).

### SDS-PAGE and western blot analysis

SDS-PAGE and western blot analysis was performed as previously described (Hoye et al., 2022). Briefly, E12.5 or E14.5 microdissected cortices were flash frozen in liquid nitrogen, then stored at −80°C until needed. Lysates were prepared using RIPA buffer (Pierce, 89900) supplemented with protease inhibitors (Sigma-Aldrich, 78429). Samples were triturated and spun for at 20,000 ***g*** for 15 min at 4°C, then quantified using a BCA quantification kit (Thermo Fisher Scientific, 23277). Equal amounts of protein were mixed with 2× Laemmli sample buffer (Bio-Rad, 1610737) supplemented with 5% β-mercaptoethanol, boiled at 95°C for 10 min, and then loaded and run on 4-20% polyacrylamide gels (Bio-Rad, 4568094). Semi-dry transfers were performed using the Bio-Rad Trans-Blot Turbo system (Bio-Rad, 1704150, 1704157). Membranes were rinsed briefly in TBST (0.1% Tween 20 in 1× TBS), blocked in 5% milk in TBST for 1 h at room temperature, then incubated at 4°C overnight in primary antibodies diluted in block. Primary antibodies used include rabbit anti-DDX3X (Sigma-Aldrich, HPA001648, RRID:AB_1078635, 1:1000) and mouse anti-β-actin (ACTB; Santa Cruz sc-47778, 1:250). Membranes were washed 5× with TBST, then incubated with horseradish peroxidase (HRP)-conjugated secondary antibodies diluted in TBST for 1 h at room temperature. Secondary antibodies used include anti-rabbit HRP (Thermo Fisher Scientific, 32430, RRID:AB_2534782, 1:2000) and anti-mouse HRP (Thermo Fisher Scientific, A16110, RRID:AB_1185566, 1:2000). Membranes were again washed 5× with TBST, then developed using a 4:1 dilution of Pierce ECL Western Blotting Substrate (Thermo Fisher Scientific, 32106):SuperSignal West Femto Maximum Sensitivity Substrate (Thermo Fisher Scientific, 34094) and imaged on a ChemiDoc XRS+ (Bio-Rad). Densitometry analysis was performed as previously described (Alsina et al., 2024).

### Figure preparation

The following illustrations from the National Institute of Allergy and Infectious Diseases NIH BioArt Source were used in generating figures. Fig. 1A: ribosome large subunit, bioart.niaid.nih.gov/bioart/450; ribosome small subunit, bioart.niaid.nih.gov/bioart/451; ssRNA brush, bioart.niaid.nih.gov/bioart/453. Fig. 1B: DNA, bioart.niaid.nih.gov/bioart/123; ssRNA brush, bioart.niaid.nih.gov/bioart/453. Fig. 6A and Fig. 7A: next-gen sequencer, bioart.niaid.nih.gov/bioart/386.

## Supplementary Material

10.1242/dmm.052498_sup1 Supplementary information

Table S1.

Table S2.

Table S3.

Table S4.

Table S5.

Table S6.
