# Supplementary material for: Impaired cortical development and translational control in a missense mouse model of DDX3X syndrome
Source: Dis Model Mech. 2025 Nov 28;18(11):dmm052498. doi: 10.1242/dmm.052498 (PMC12690540; doi:10.1242/dmm.052498)
Supplement: Supplementary information [file dmm-18-052498-s1.pdf]

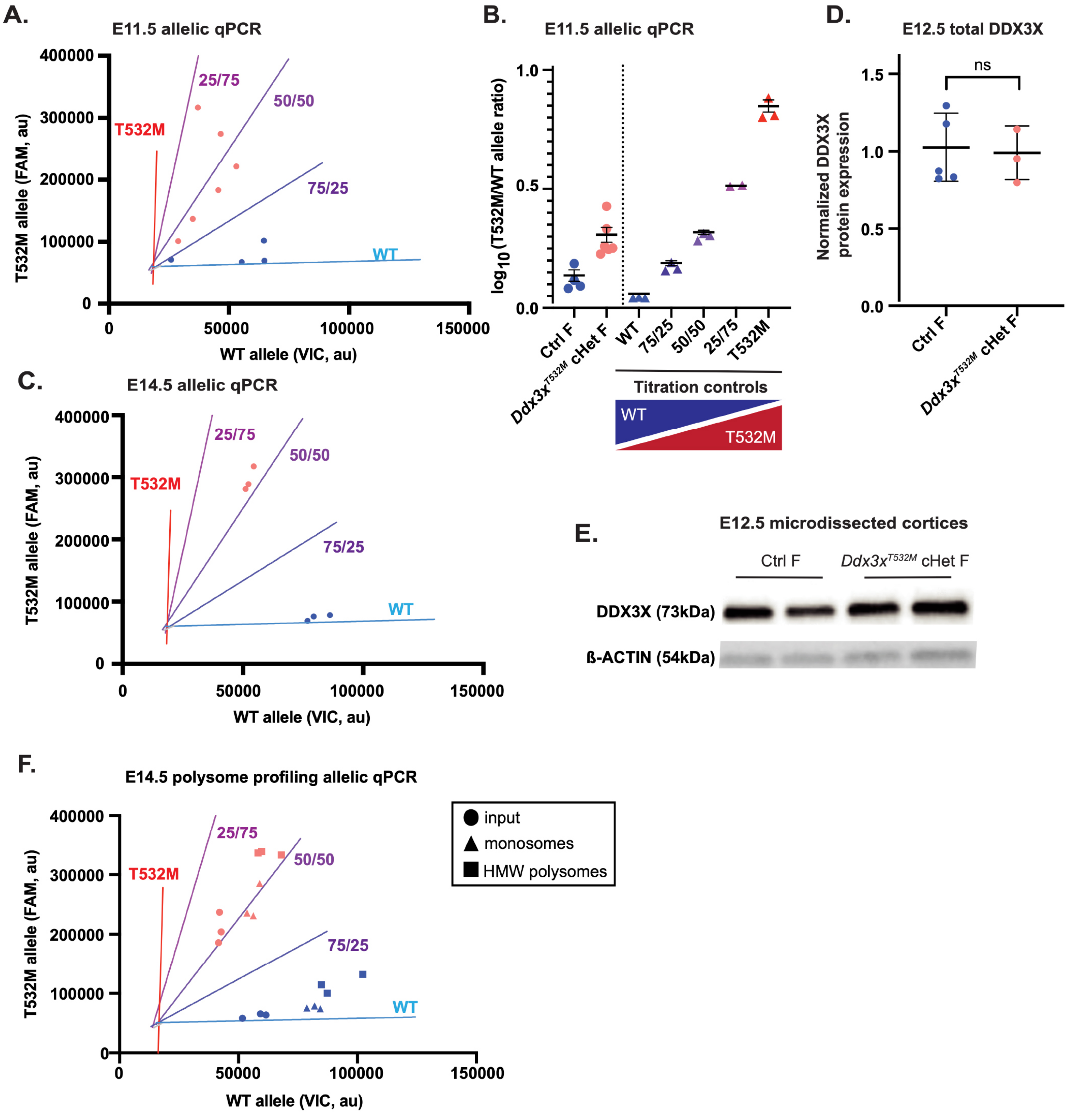

Disease Models & Mechanisms • Supplementary information

Fig. S1. Additional validation of conditional knock-in model for *Ddx3x^{T532M}*.

A) Allelic qPCR of FAGS-isolated Cre^{+} cells at E11.5. Each line is the best fit line of three titration controls (two titration controls for 25/75 only), assayed in triplicate, of denoted ratio of WT/ T532M alleles. Each point is representative of a single embryo, assayed in triplicate; points are color-coded by genotype as follows. N = 4 *Emx1*-Cre*;Rosa263^{Ai14} ;Ddx3x$^{+/+}$* (Ctrl, control) female embryos (blue), 6 *Emx1-Cre;Rosa26^{Ai14} ;Ddx3x$^{T\ 532Mlox/+}$* cHet females (salmon) from 2 litters. qPCR was performed twice. **B)** Allelic qRT-PCR analysis at E11.5 plotted as the ratio of the two alleles and normalized to 50:50 T532M/WT titration controls. Each point is representative of a single embryo, assayed in triplicate. Points are color-coded by genotype. N = 4 *Emx1-C re; Rosa26^{Ai14} ;0dx3x$^{+/+}$* control female embryos (blue), 6 *Emx1*-Cre*;Rosa26$^{Ai1\ 4}$;Ddx3x$^{T532M\ lox/+}$* cHet female embryos (salmon) from 2 litters. qPCR was performed twice . **C)** Allelic qPCR of FAGS-isolated Cre+ cells at E14.5. Each line is the best fit line of 2-3 titration controls (two titration controls for 25/75 only), assayed in triplicate, of denoted ratio of WT/T532M alleles. Each point is representative of a single embryo, assayed in triplicate; points are color-coded by genotype as follows. N = 3 *Emx1*-Cre*;Rosa26^{Ai14} ;Ddx3x$^{+/+}$* control female embryos (blue), 3 *Emx1*-Cre*;Rosa26^{Ai14} ;Ddx3x$^{T532M\ lox/+}$* cHet female embryos (salmon) from 3 litters. qPCR was performed twice. **D)** Quantification of E12.5 relative DDX3X protein expression. Each point represents an embryo. Points are color-coded by genotype. N = 5 (control F, blue), 3 *(Ddx3x$^{T\ 532M}$* cHet F, salmon) from 2 litters. **E)** Representative western blot for total DDX3X, with ACTB as a loading control, in E12.5 microdissected cortices of denoted genotypes. Western results representative of two independent western blot experiments.

F) Allelic qPCR of total RNA (circles), monosome-associated RNA (triangles), and polysome-associated RNA (squares) from E14.5 *Emx1-Cre;Rosa26^{Ai14} ;Ddx3x$^{+/+}$* control female (blue) and *Emx1*-Cre*;Rosa26^{Ai14};Ddx3x$^{T532M\ lox/+}$* cHet (salmon) female cortices. Each point represents a sample; each sample consists of cortices pooled from two embryos of the same genotype. N = 3 samples/genotype from 5 litters. Each line is the best fit line of three titration controls, assayed in triplicate, of denoted ratio of WT/T532M alleles. qPCRs were performed twice. Significance assessed by Student's unpaired, two-tailed t-test (D). ns, not significant. Error bars, mean ± SD.

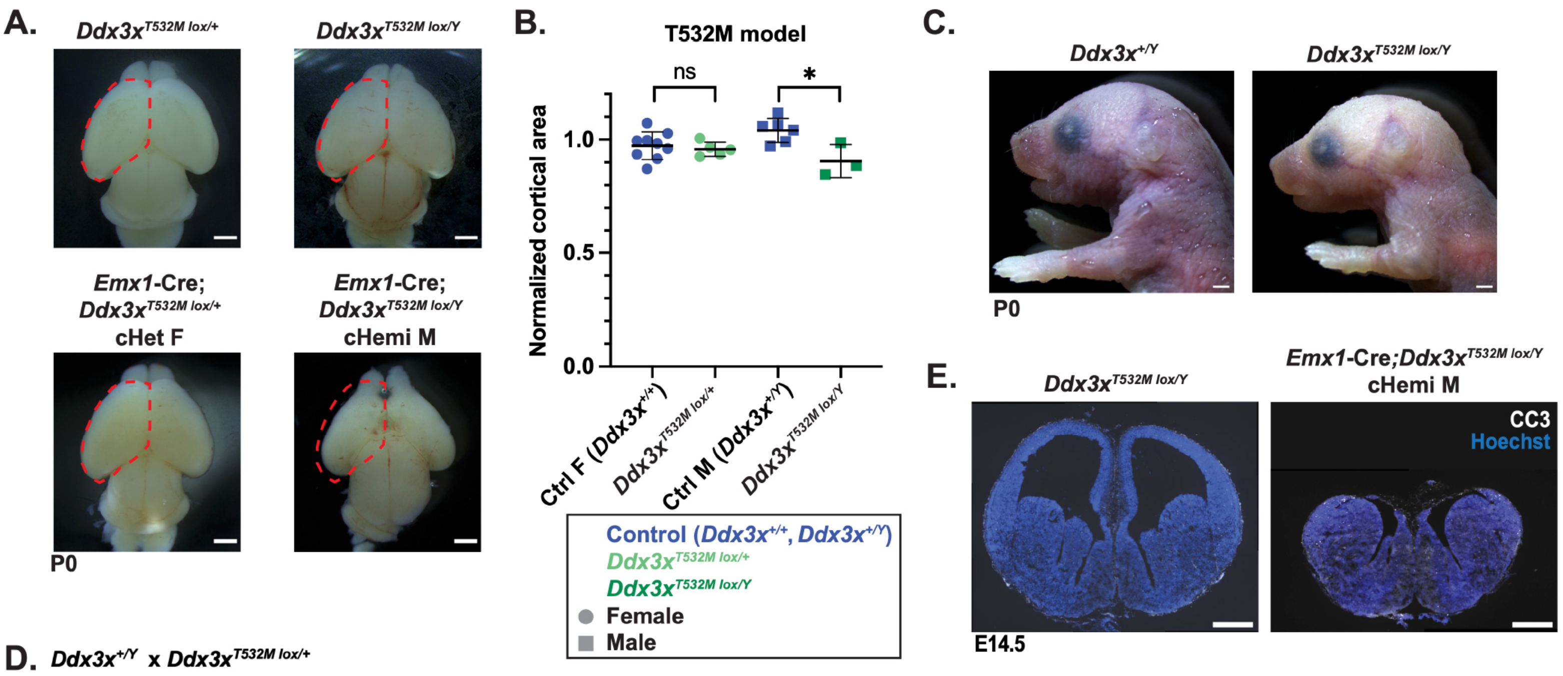

D. *Ddx3x*$^{+/Y}$ x *Ddx3x*$^{T532M\ lox/+}$

Age	*Ddx3x*$^{+/+}$	*Ddx3x*$^{+/Y}$	*Ddx3x*$^{T532M\ lox/+}$	*Ddx3x*$^{T532M\ lox/Y}$	Chi-square test
P12-P14	38% (141)	35% (129)	25% (94)	2% (6)	****
P0	28% (9)	34% (11)	28% (9)	9% (3)	ns
E14.5	30% (8)	33% (9)	22% (6)	15% (4)	ns

Fig. S2. Microcephaly and lethality phenotypes of *DdxJ x*$^{T532M\ iox/Y}$ male mice.

A) Representative images of brains from a *Ddx3x*$^{T532M\ lox/+}$ female, *Ddx3x*$^{T532M\ lox/Y}$ male, *Emx1*-Cre*; Ddx3x*$^{T532M\ l0x/+}$ cHet female, and an *Emx1-Cre;Ddx3x*$^{T532M\ lox/Y}$ cHemi male, demonstrating the specificity of the microcephaly phenotype to *Emx1-Cre;Ddx3x*$^{T532M\ lox/Y}$ cHemi males. Red trace outlines cortical area of *Ddx3x*$^{T532M\ lox/+}$ brain and is superimposed onto other genotypes. **B)** Quantification of cortical areas as in Fig. 3B in control (ctrl) *Ddx3x*$^{+/Y}$ males, control *Ddx3x*$^{+/+}$ females, *Ddx3x*$^{T532M\ lox/+}$ females and *Ddx3xr*$^{T532M\ lox/Y}$ males. Each point denotes the average of quantifications of both cortices per a single embryo. Squares represent males, and circles represent females; points are color-coded by genotype. N = *5Ddx3x*$^{+/Y}$, 9*Ddx3x*$^{+/+}$, 3*Ddx3x*$^{T532M\ lox/+}$, from 4 litters. **C)** Representative images of PO pups of denoted genotypes. **D)** Quantification and Chi-squared analyses of offspring genotypes across developmental stages as denoted from the cross of a *Ddx3x*$^{+/Y}$ male with a *Ddx3x*$^{T532M\ lox/+}$ cHet female. Percentage recovery for each genotype/stage is listed first, followed by the total number of individuals in parenthesis. **E)** Cleaved caspase 3 (CC3, white) and Hoechst (blue) immunofluorescence in coronal cryosections from embryonic brains of denoted genotypes at E14.5, demonstrating specificity of cortical reduction to *Emx1*-Cre*;Ddx3x*$^{T532M\ lox/Y}$ cHemi males. CC3 staining was performed twice. Statistics are one-way ANOVA with multiple comparisons (B); any unshown comparisons are not significant. Statistics are Chi-squared test (D). *, $p < 0.05$; ****, $p < 0.0001$; ns, not significant. Scale bars: 0.01 cm (A); 1 mm (C); 0.02 cm (E). Error bars, mean ± SD.

Disease Models & Mechanisms • Supplementary information

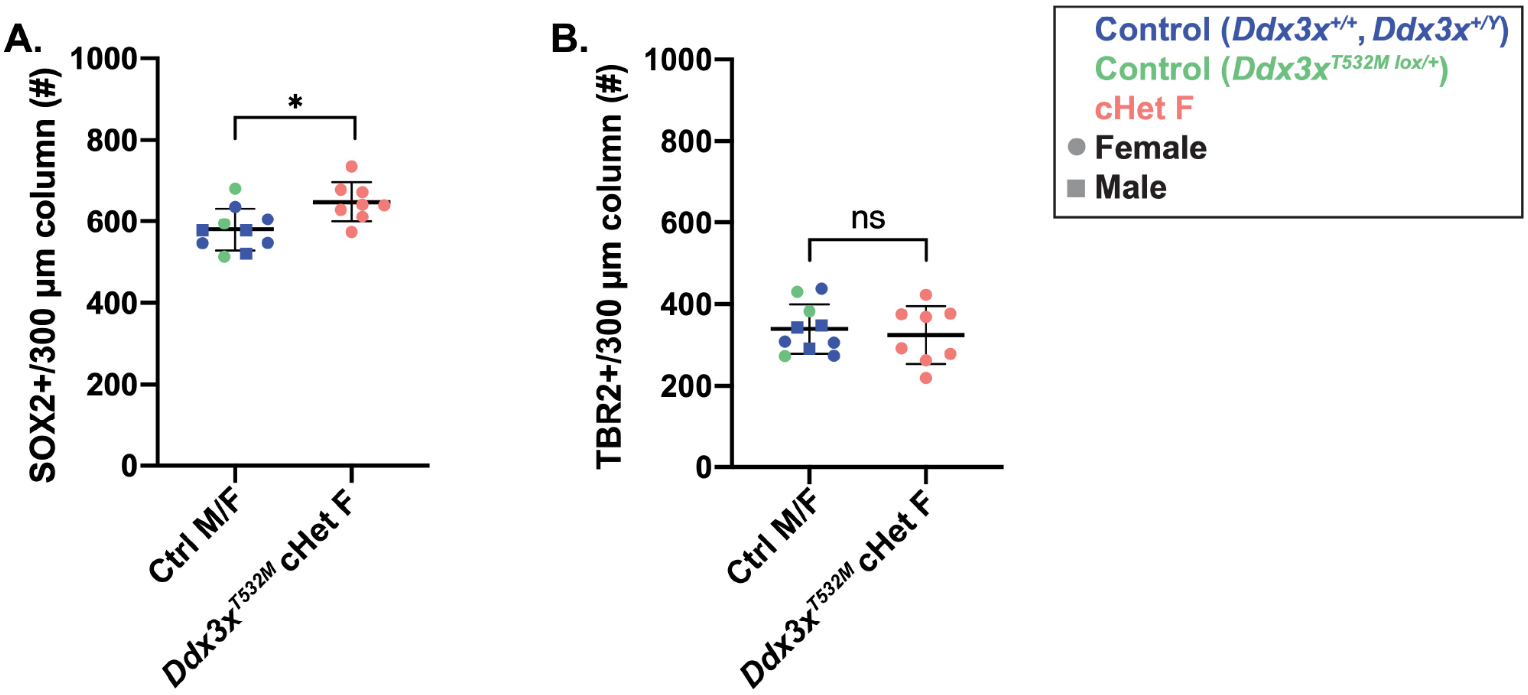

Fig. S3. *Ddx3x*^{T532M} *cHet* females have a mild increase in radial glial cells, but not intermediate progenitors.

A) Quantifications of SOX2+ radial glial cells per 300 µm column at E14.5. **B)** Quantification of TBR2+ intermediate progenitors per 300 µm column at E14.5. Each point is the average of 2- 3 quantified coronal sections per single embryo. Squares represent males, and circles represent females; points are color-coded by genotype. N = 10 (Ctrl, control M/F, including 7 *Ddx3x*$^{+/+}$ or *Ddx3x*$^{+/Y}$, with or without *Emx1*-Cre [blue], and 3 *Ddx3x*$^{T532M\ lox/+}$ F [green]); 8 *Emx1-Cre;Ddx3x*$^{T532M\ lox/+}$ cHet F (salmon), from 7 litters. Staining experiment was repeated twice. Statistics are Student's unpaired, two-tailed t-tests (A- B). *, $p < 0.05$; ns, not significant. Error bars, mean ± SD.

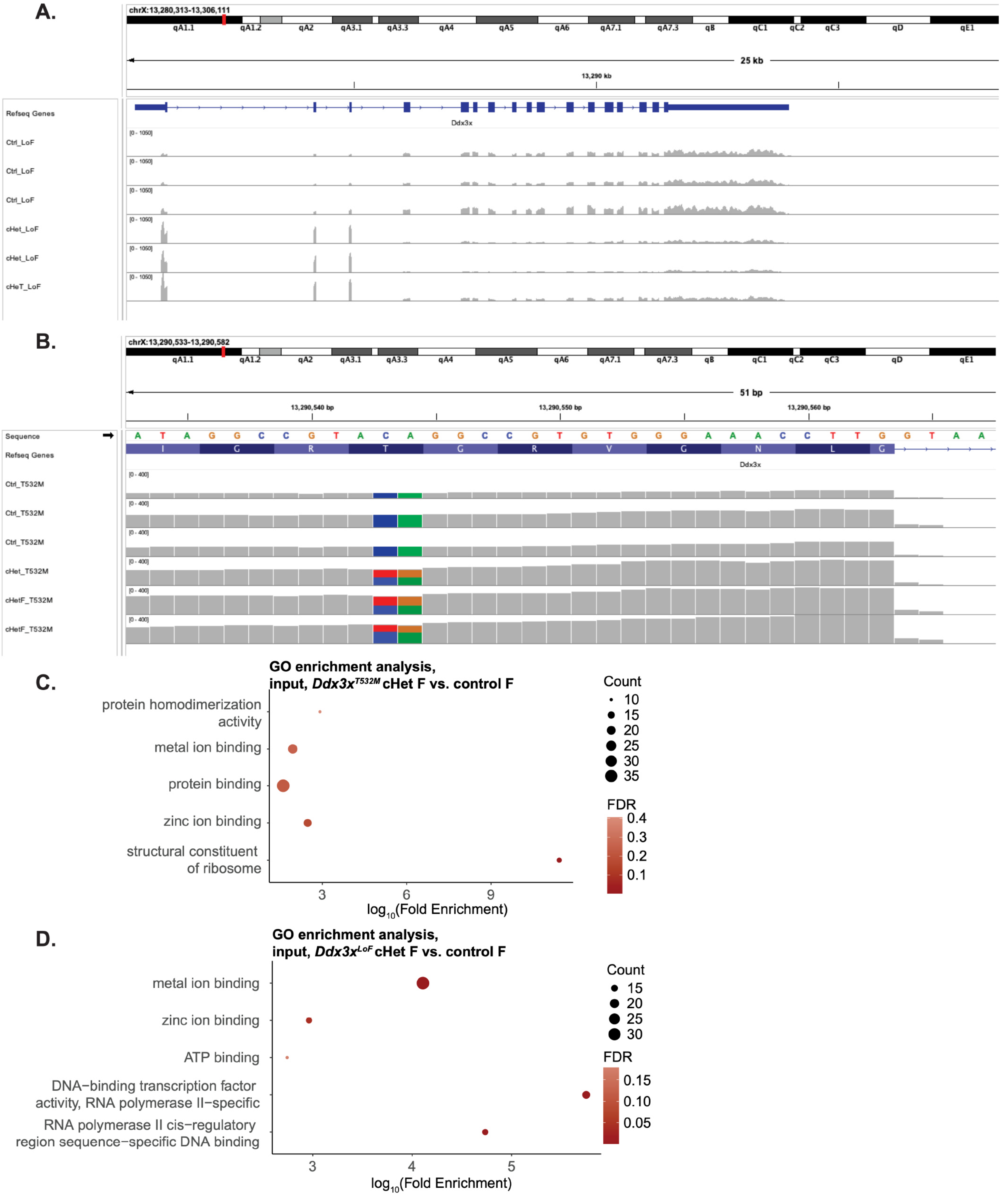

Fig. S4. RNAseq and polysome fractionation validate genetic models and reveal discrete categories of gene regulation.

A) Alignments of *Emx1*-Cre;*Ddx3x*$^{lox/+}$ cHet F and *Ddx3x*$^{lox/Y}$ control female RNAseq reads at *DDX3X* locus, demonstrating dropoff of reads in cHet Fs after exon 3, the final exon remaining upon Cre recombination of a floxed allele in this line. **B)** Alignments of *Emx1*-C re;*Ddx3x*$^{T532M\ lox/+}$ cHet and *Emx1*-Cre;*Ddx3x*$^{+/+}$ control female RNAseq reads at *DDX3X* locus, with calls at T532 color-coded by nucleotide. **C)** Molecular function GO analysis for differentially expressed genes in total RNA samples from *Emx1*-C re;*Ddx3xr*$^{T532M\ lox/+}$ cHet and *Emx1*-Cre;*Ddx3x*$^{+/+}$ control females. **D)** Molecular function GO analysis for differentially expressed genes in total RNA samples from *Emx1*-Cre;*Ddx3x*$^{lox/+}$ cHet and *Ddx3x*$^{lox/Y}$ control females.

Disease Models & Mechanisms • Supplementary information

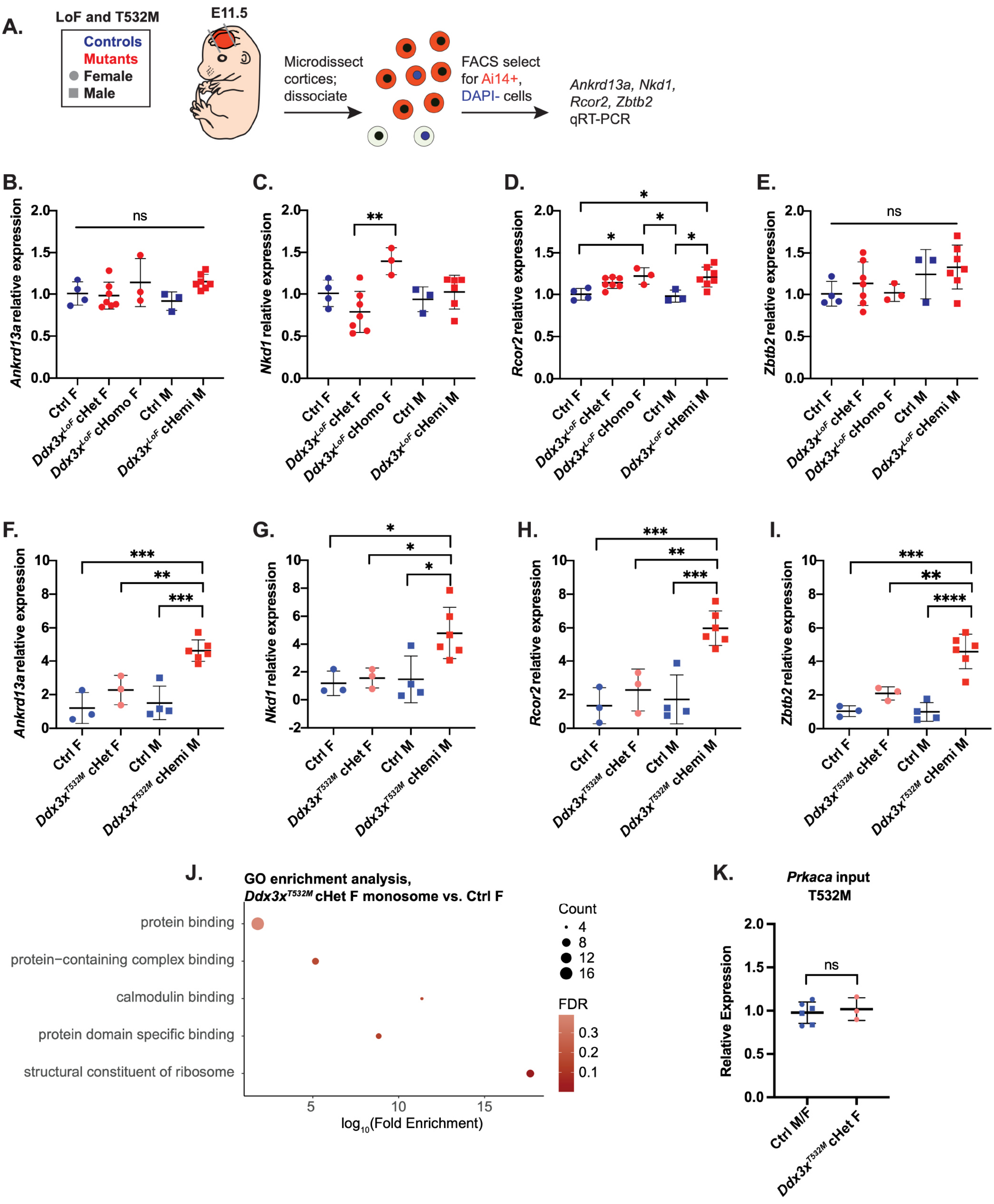

Fig. S5. Validation of RNAseq targets at E11.5 underscores sexually dimorphic phenotypes in both conditional loss-of-function and conditional knock-in models.

A) Schematic of E11.5 FACS experiment across both *Ddx3x*^{T532M} and *Ddx3x*^{LoF} conditional models. **B-E)**

Disease Models & Mechanisms • Supplementary information

qRT-PCR results for *Ankrd13a* **(B),** *Nkd1* **(C)**, *Rcor2* **(D)**, and *Zbtb2* **(E)** in E11.5 FACS samples from mice of denoted genotypes from the *Ddx3x* loss-of-function conditional model. For LoF model (B-E), N = 4 *Emx1*-Cre*;Rosa26*^{Ai14}*;Ddx3x*$^{+/+}$ control (ctrl) females; 3 *Emx1*-Cre*; Rosa26*$^{Ai/14}$*;Ddx3x*$^{+/Y}$ control males; 7 *Emx1*-Cre*; Rosa26*^{Ai14}*;Ddx3x*$^{lox/+}$ cHet females; 7 *Emx1*-Cre*;Rosa26*^{Ai14}*;Ddx3x*$^{+/Y}$ cHemi males; 3 *Emx1*-Cre*;Rosa26*^{Ai14}*; Ddx3x*$^{lox/lox}$ cHomo females from 10 litters. **F-I)** qRT-PCR results for *Ankrd13a* **(F),** *Nkd1* **(G)**, *Rcor2* **(H),** and *Zbtb2* **(I)** in E11.5 FACS samples from mice of denoted genotypes from the *Ddx3x*^{T532M} conditional model. For T532M model (F-I), N =3 *Emx1*-Cre*;Rosa26*^{Ai14}*;Ddx3x*$^{+/+}$ control females; 4 *Emx1*-Cre*;Rosa26*^{Ai14}*;Ddx3x*$^{+/Y}$ control males; 3 *Emx1*-Cre*; Rosa26*^{Ai14}*;Ddx3x*$^{T532M\ l0x/Y}$ cHet females; 6 *Emx1*-Cre*;Rosa26*^{Ai14}*;Ddx3x*$^{T532M\ l0x/y}$ cHemi males from 2 litters. **J)** Molecular function GO analysis for monosome-enriched genes from *Emx1*-Cre*;Ddx3x*$^{T532M\ l0x/+}$ cHet and *Emx1*-Cre*;Ddx3x*$^{+/+}$ control females. **K)** qRT-PCR for *Prkaca* in input E14.5 RNA samples of denoted genotypes. N = 3 samples/genotype from 5 litters. Squares represent males, and circles represent females; points are color-coded by genotype. All qRT-PCR results are first normalized to loading control *β-actin,* then to control females of the appropriate model. Each point represents a single embryonic sample (B-1) or a single sample (derived from cortices of a pooled pair of like-genotype embryos, as in Fig 6A, assayed in technical triplicate) (K). qPCRs were performed once. Statistics are one-way ANOVA with multiple comparisons (B-1). Any comparison not shown is not significant; ns label is only used in the case where no comparison within an experiment is significant. Statistics for (K) are Student's unpaired, two-tailed t-test. *, p < 0.05; **, p < 0.005; ***, p < 0.0005; ****, p < 0.0001; ns, not significant. Error bars, mean ± SD.

Table S1. RNAseq differential expression analysis of *Ddx3x*^{T532M} cHet female cortices vs control female cortices.

Available for download at
https://journals.biologists.com/dmm/article-lookup/doi/10.1242/dmm.052498#supplementary-data

Table S2. RNAseq differential expression analysis of *Ddx3x*^{LoF} cHet female cortices vs control female cortices.

Available for download at
https://journals.biologists.com/dmm/article-lookup/doi/10.1242/dmm.052498#supplementary-data

Table S3. Monosome enrichment analysis of *Ddx3x*^{T532M} cHet female cortices vs control female cortices.

Available for download at
https://journals.biologists.com/dmm/article-lookup/doi/10.1242/dmm.052498#supplementary-data

Table S4. Polysome enrichment analysis of *Ddx3x*^{T532M} cHet female cortices vs control female cortices.

Available for download at
https://journals.biologists.com/dmm/article-lookup/doi/10.1242/dmm.052498#supplementary-data

Table S5. Monosome enrichment analysis of *Ddx3x*^{LoF} cHet female cortices vs control female cortices.

Available for download at
https://journals.biologists.com/dmm/article-lookup/doi/10.1242/dmm.052498#supplementary-data

Table S6. Polysome enrichment analysis of *Ddx3x*^{LoF} cHet female cortices vs control female cortices.

Available for download at
https://journals.biologists.com/dmm/article-lookup/doi/10.1242/dmm.052498#supplementary-data
